# Highest densities of mountain hares (*Lepus timidus*) associated with ecologically restored bog but not grouse moorland management

**DOI:** 10.1002/ece3.8744

**Published:** 2022-03-31

**Authors:** Carlos P. E. Bedson, Philip M. Wheeler, Neil Reid, Wilson Edwin Harris, David Mallon, Simon Caporn, Richard Preziosi

**Affiliations:** ^1^ 5289 Department of Natural Sciences Manchester Metropolitan University Manchester UK; ^2^ School of Environment, Earth and Ecosystem Sciences The Open University Milton Keynes UK; ^3^ Institute of Global Food Security (IGFS) School of Biological Sciences Queen’s University Belfast Belfast UK; ^4^ Department of Agriculture and Environment Harper Adams University Newport UK; ^5^ Faculty of Science and Engineering University of Plymouth Devon UK

**Keywords:** blanket bog, distance sampling, grouse moor, habitat degradation, landscape restoration, monitoring, mountain hare, peatland

## Abstract

Over the last 20 years, ecological restoration of degraded habitats has become common in conservation practice. Mountain hares (*Lepus timidus scoticus*) were surveyed during 2017–2021 using 830 km of line transects in the Peak District National Park, England. Historically degraded bog areas were previously reported having low hare numbers. Following bog restoration, we found hare densities of 32.6 individuals km^−2^, notably higher than neighboring degraded (unrestored) bog with 24.4 hares km^−2^. Hare density on restored peatland was 2.7 times higher than on bogs managed for grouse shooting at 12.2 hares km^−2^ and 3.3 times higher than on heather moorland managed for grouse shooting at 10.0 hares km^−2^. Yearly estimates varied most on habitats managed for grouse, perhaps indicative of the impact of habitat management, for example, heather burning and/or possible hare culling to control potential tick‐borne louping ill virus in gamebirds. Acid grassland used for sheep farming had a similar density to grouse moorland at 11.8 hares km^−2^. Unmanaged dwarf shrub heath had the lowest density at 4.8 hares km^−2^. Hare populations are characterized by significant yearly fluctuations, those in the study area increasing by 60% between 2017 and 2018 before declining by *ca*. 15% by 2020 and remaining stable to 2021. During an earlier survey in 2002, total abundance throughout the Peak District National Park was estimated at 3361 (95% CI: 2431–4612) hares. The present study estimated 3562 (2291–5624) hares suggesting a stable population over the last two decades despite fluctuations likely influenced by weather and anthropogenic factors. Mountain hares in the Peak District favored bog habitats and were associated with restored peatland habitat. Wildlife management should be cognizant of hare density variation between habitats, which may have implications for local extinction risk.

## Graphical abstract

During 2017–2021 we surveyed mountain hares on different habitat classes using distance sampling methods. Our findings showed the highest densities of mountain hares on restored peatland habitats. Areas of peatland bog and heather moorland, managed for grouse shooting, hosted intermediate densities of mountain hares.

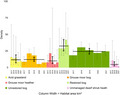

## 1 INTRODUCTION

Across the world, many ecosystems are suffering anthropogenic damage with wide‐ranging impacts (IPBES, 2019). Among these are peatlands, wetland ecosystems where decomposing vegetation has taken thousands of years to accumulate as peat layers. These are often vulnerable to human activities (e.g., cutting, grazing, burning, and indirect erosion) and sensitive; their replacement may require millennia (Page & Baird, 2016; Yu et al., 2016). In the northern hemisphere, peatlands experience cold‐wet climates, providing the conditions for peat layer development. Peatland habitat stores approximately 50% of total global soil carbon storage (Evans et al., 2006), while hosting environmentally sensitive plants and animals of high conservation importance. Across Europe, many peatlands are degraded (Urak et al., 2017) and substantial funds (e.g., ~ **€**167 m in EU Life projects) have been invested in peatland restoration in recent decades, recognizing its importance for carbon sequestration, water retention, and biodiversity (Andersen et al., 2017).

The South Pennine Moors contains 650 km^2^ of UK upland peatland distribution (Bonn et al., 2009; JNCC, 2015) and received Special Areas of Conservation (SAC) designation in 2005 for its unique upland plant community and population of breeding waders (Natural England, 2005, 2019). This area features peatlands which have suffered extensive human‐caused degradation (Evans, 2009). Over the last two centuries, atmospheric pollutant deposition from the surrounding industrial cities including Sheffield and Manchester led to local soil acidification and loss of sphagnum, severely harming vegetation, leaving bare peat and extensive gully erosion (Alderson et al., 2019; Andersen et al., 2017; Natural England, 1993; Tallis, 1997, 1998). Within the SAC are ~350 km^2^ of grouse moor estates practicing rotational heather burning and predator management, creating an ecosystem supporting red grouse (*Lagopus lagopus*) for shooting (Phillips, 2012; Sotherton, 2009). There are also areas, which have seen extensive sheep (*Ovis aries*) overgrazing, where former upland dry heath has transitioned to acid grassland (Anderson & Yalden, 1981). The frequency of accidental or deliberate wildfires has also increased (McMorrow et al., 2009). All these anthropogenic mechanisms have been implicated in causing extensive moorland degradation, precipitating much loss of diversity of flora and fauna (Anderson & Shimwell, 1981; Pearce‐Higgins et al., 2006; Sim et al., 2005; Tallis, 1998; Thompson et al., 1995; Tucker, 2003). Recent evaluation of habitat conditions for the South Pennine Moors SAC rated the area as 99% “unfavorable‐recovering” or “unfavorable‐no change” (Natural England, 2021).

From 2003, a well‐funded (~ **€**35 m) restoration program managed by the Moors for the Future Partnership commenced in the South Pennine Moors SAC (Alderson et al., 2019; Bedson *in litteris*.). Conservation measures included fencing out grazing animals, reduced burning and trampling, and removal of species, for example, *Molinia*. Hydrology was re‐established with gully blocking. Bare peat was restored with netting, fertilizers, liming, mulches and reseeding and replanting with grasses, rushes, mosses, dwarf shrubs, heather, and eventually *Sphagnum* moss (Alderson et al., 2019; Buckler et al., 2013). Many bare peat areas recovered their vegetation (Alderson et al., 2019). However, little was known about the effects on wildlife (Andersen et al., 2017; Shepherd et al., 2013).

The mammal species mountain hare (*Lepus timidus scoticus*) has been regarded as a useful habitat quality indicator (JNCC, 2008). This cold‐adapted lagomorph is associated with UK upland peatlands, playing an important role as both herbivore and prey for foxes (*Vulpes vulpes*), stoats (*Mustela erminea*), and raptors (Yalden, 2009). Elsewhere across Europe and Asia, mountain hares occupy a range of elevations, inhabiting tundra, taiga, boreal forests, bogs, and grasslands at low population densities of 1–6 individuals km^−2^, though higher, on some Baltic islands (25–60 km^−2^) and far east Russia (200–400 km^−2^) (Angerbjorn & Flux, 1995). Mountain hare density is high (50–200 km^−2^) on managed grouse moor habitat in Scotland. Rotational heather burning provides early‐stage heather favored by hares (Flux, 1962; Hewson, 1976, 1989; Savory, 1986). Predator control (e.g., shooting or trapping of foxes, stoats, weasels (*M*. *nivalis*), corvids, or historically, raptors) to protect grouse was also purported to support hares (Patton et al., 2010; Stoddart & Hewson, 1984). However, many grouse moor estates also shot hares for sport (Hesford et al., 2020; Patton et al., 2010). More recently, culls were organized to substantially reduce hare numbers, on the grounds that hares transmit ticks carrying louping ill virus to grouse (Patton et al., 2010; Watson & Wilson, 2018); although evidence of mountain hares being a principal vector for this disease transmission is ambiguous (Harrison et al., 2010). Annual hunting of hares until 2016 averaged 39,000 individuals (95% CI: 16,000–70,000) (Aebischer, 2019). The recent assessment under Article 17 1992 EC Habitats and Species Directive described UK mountain hare status as “deteriorating” and “unfavorable‐inadequate” (JNCC, 2019). Populations cycle with up to 80% amplitude, confounding conservation monitoring (Newey, Dahl, et al., 2007). Yet, the central tendency of census estimates has decreased from 350,000 (95% CI: 93,000–709,000) (JNCC, 2007) to 132,000 individuals (95% CI: 79,000–516,000) (JNCC, 2019).

In England, mountain hares became extinct around 6000 bp and were reintroduced to areas of the South Pennines Moors lying within the present‐day Peak District National Park, by landowners with sporting interests in the 1870s (Harris & Yalden, 2008). From the 1970s, studies described a small, stable population of ~1000 individuals (Mallon, 2001; Yalden, 1971, 1984). The last field study estimated ~10,000 individuals, inconsistent with previous research (Mallon et al., 2003). The most recent estimate was 2500 individuals (Mathews et al., 2018). Mountain hares were associated with mixed *Calluna*/*Eriophorum* areas or *Calluna* areas on grouse moors (Mallon et al., 2003; Yalden, 1971), and there were concerns about the persistence of these habitats (JNCC, 2007).

The aim of this research was to estimate mountain hare densities in different upland habitats. We surveyed mountain hares over 5 years and evaluated evidence whether habitat restoration and/or grouse moor management was concomitant with high hare population density. In 1 year, we also surveyed the whole National Park to report overall mountain hare abundance. This research was intended to accomplish investigations recommended by the UK Biodiversity Action Plan (JNCC, 2008) and to inform future conservation status assessments.

## 2 MATERIALS AND METHODS

### 2.1 Study area

**FIGURE 1:**
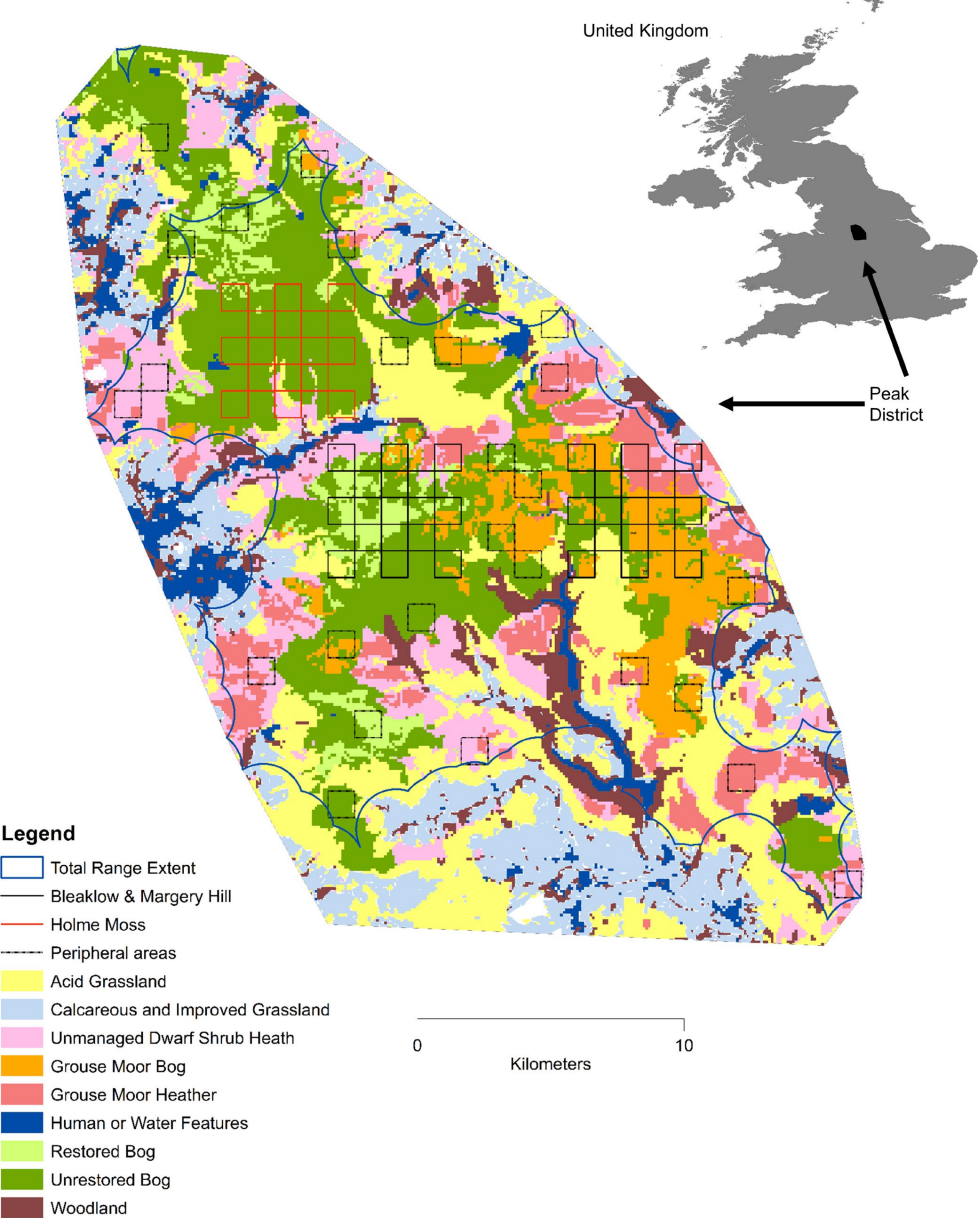
Map of study area. The locations of 10 years of BRC mountain hare records informed the minimum convex polygon, being the outer shape. The extent of hares for abundance projection was the alpha hull shape, shown by the blue line and also the survey areas. The survey transects are shown for Bleaklow and Margery Hill (black squares); Holme Moss (red squares); and peripheral areas (dotted squares). Legend shows habitat classes. Inset map shows location of Peak District in the United Kingdom. Peak District map origin is British National Grid Reference SK Easting 390000 Northing 370000. North at top

Fieldwork was conducted on upland habitats in the Peak District National Park, lying within the South Pennine Moors SAC (Figure 1). These uplands are underlain by acidic gritstone and shale rocks forming hills up to ~630 m. The annual average temperature is 10.3°C and precipitation 1025 mm, creating a wet substrate on hill tops (UK Met Office, 2020). The hills are covered with peat, up to 2 m deep (Anderson & Shimwell, 1981). The study extent was informed by UK Biological Record Centre (BRC) mountain hare observations (See Acknowledgments) for the period 1998–2018, eliciting 8666 records. From these, we mapped a minimum convex polygon 610 km^2^ constituting the observed mountain hare range in our study area (Figure 1).

### 2.2 Habitat classes

**FIGURE 2:**
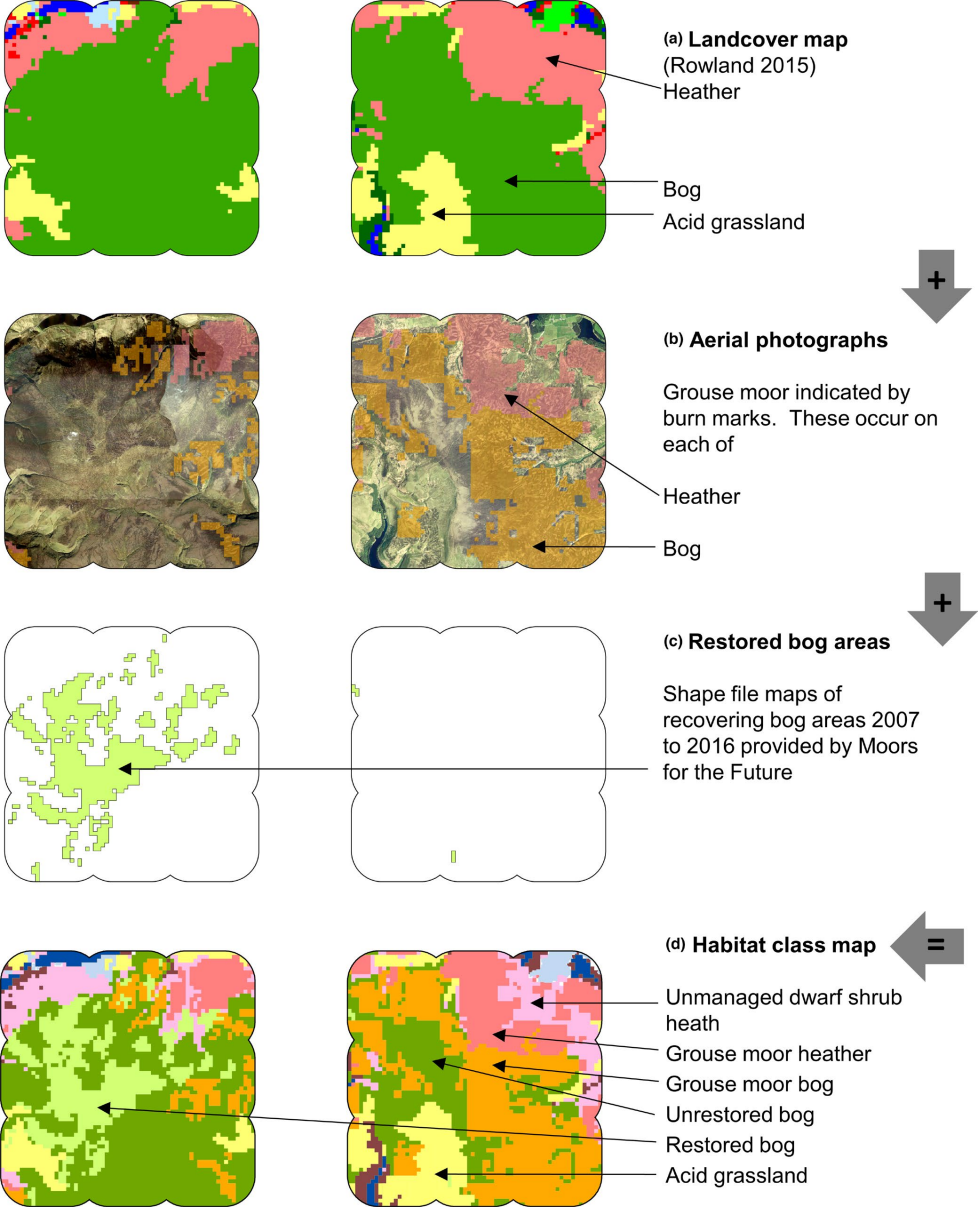
Step‐by‐step construction of habitat class map for surveyed extent (5 × 5 km with 800 m buffer) with 1‐ha pixel, for each of Bleaklow (left) and Margery Hill (right) (British National Grid origin SK Easting 408000 Northing 394000). Map (a) shows landcover classification system of Rowland et al. (2017), which is used as starting point. Map (b) Aerial photographs are assessed and any with burn mark within any hectare denoted as either grouse moor bog or grouse heather, referencing the underlying landcover determined by Rowland et al. (2017). Map (c) Shapefiles provided by Moors for the Future, showing recovering bog areas which received treatment up to 2016. Map (d) The final map with all habitat classes pertinent to mountain hares. Any heather without burn mark is, therefore, regarded as unmanaged dwarf shrub heath

We developed a habitat classification map by layering several data sources and mapping with a 1‐ha scale cell grid (i.e., 100 cells km^−2^)in ArcGIS (ESRI USA) (Figure 2). Habitat classes pertaining to mountain hare occupancy were acid grassland, upland dwarf shrub heath, and wet upland blanket bog (Jackson, 2000; Natural England, 2005, 2019), with extent informed by the UK landcover map (Rowland et al., 2017). Other habitats had very few mountain hare records and were deemed irrelevant.

Acid grassland occurred at 300–550 m elevation, comprising a broad habitat type of calcifugous swards dominated by grasses (*Festuca ovina*, *Nardus stricta*), sedges, and herbs on lime‐deficient soils, pH <5.5 (Jackson, 2000; Rowland et al., 2017). In winter (when mountain hares were surveyed), grasses and bracken (*Pteridium*) were senescent; *Calluna* reaching to 80 cm height, *Juncus* and *Molinia* reach 120 cm height (Stace, 2010). These areas were used for sheep rearing.

Blanket bog comprised ombrotrophic wetlands supporting vegetation forming deep peat overlaying hill plateaus (Natural England, 1993). *Eriophorum vaginatum* was dominant, with *Sphagnum* mosses and bog pools present (Anderson & Shimwell, 1981; IUCN, 2014; Natural England, 2005; Rowland et al., 2017). These areas had been extensively eroded (Natural England, 1993). We subdivided the blanket bog landcover area to “grouse moor bog,” “restored bog,” and “unrestored bog” described below. Upland dry heath occupied lower slopes of moors on mineral soils or shallow peat areas, strongly dominated by *Calluna vulgaris*‐*Deschampsia flexuosa* and *C*. *vulgaris*‐*Vaccinium myrtillus* heath. (Anderson & Shimwell, 1981; Elkington et al., 2001; Natural England, 2005; Rowland et al., 2017) and we subdivided this to “grouse moor heather” or “unmanaged dwarf shrub heath” described below.

To identify grouse moor areas, we followed methods from Yallop et al. (2006) and assembled a mosaic of 1:500 scale aerial images dated for 2018 (Digimap, 2019). Any 1‐ha cell showing a burn or mowed patch was designated as “grouse moor bog” or “grouse moor heather” depending on underlying landcover (Rowland et al., 2017). Grouse moors featured rotationally burned areas, shooting butts, grit trays, quad bike tracks, and predator (corvid and mustelid) traps. “Grouse moor bog” at elevations 350–530 m was wet heath overlying deep peat with eroded gullies, *Calluna*, more *Eriophorum* spp. and mosses. “Grouse moor heather” at elevations 280–510 m was drier areas with shallow peat, few gullies, and extensive *Calluna* (Allen et al., 2016). Burned heather comprised different succession stages: suppressed (“pioneer”) heather, height to 15 cm; sub‐dominant heather, age to 10+years, height ~15 cm, coverage ~40%; dominant heather, age up to 25 years, height ~30–120 cm coverage, 60+% (Allen et al., 2016; Bardgett et al., 1995; Stace, 2010; Whitehead et al., 2021). Also present were *Eriophorum*, *Sphagnum* and other mosses, cross‐leaved heather *Erica tetralix*, bell heather *Erica cinerea*, bilberry *Vaccinium myrtillus,* and crowberry *Empetrum nigrum* (Bardgett et al., 1995; Whitehead et al., 2021). The Peak District was recorded with burns as 29% of total potential burn area and patch sizes 500–1000 m^2^ (Allen et al., 2016).

The remaining heather area not grouse moor was classified as “unmanaged dwarf shrub heath” at elevations 250–520 m including steep slopes and few gullies. This comprised mosaics of 70% dense/30% open heather, predominantly *Calluna* (Rowland et al., 2017), height to 120 cm (Bardgett et al., 1995; Stace, 2010). There was no predator control and few sheep.

We identified “restored bog” from shapefiles provided by the conservation partnership “Moors for the Future” (Acknowledgments), designating their recovery work to 2016. These areas measured ~20 km^2^, occurring at elevations 480–630 m and comprised previously degraded bare peat. From 2007, restoration efforts included gully blocking, fertilizer, liming, laying of jute textiles, reseeding, planting, and spreading heather brash (Alderson et al., 2019). By 2016, this work achieved 75% vegetation cover (Alderson et al., 2019); much was in lush, verdant condition. Vegetation comprised a wide variety of moorland species, which shifted frequently in composition over the space of a few meters, including *Calluna*, *Eriophorum*, and *Sphagnum* spp., shrubs (e.g., *Erica tetralix*, *E*. *cinerea*, *Rubus chamaemorus*, *Vaccinium mytrillus*, and *Empetrum nigrum*), ferns (e.g., *Oreopteris limbosperma* and *Blechnum spicant*), herbs (e.g., *Potentilla erecta*, *Viola palustris*, *Chamerion angustifolium*, and *Galium saxatile*), and mosses (e.g., *Hypnum jutlandicum* and *Polytrichum spp*). *Calluna* height was up to ~100 cm; winter grasses were senescent reaching heights ~30 cm (Stace, 2010). The extensive networks of eroded gullies were revegetated, and the water table was high (Alderson et al., 2019). There was no predator control practiced, and sheep were fenced out.

The remaining bog areas were classed as “unrestored bog” at elevations 300–630 m. These had not historically deteriorated to the point of comprising bare peat, yet nonetheless appeared ecologically impoverished, that is, “unfavorable‐recovering” condition (Natural England, 2019). They consisted mostly of extensive fields of *Eriophorum* spp. and *Molinia caerulea* grass, winter height ~30 cm, and some *Calluna* patches height ~100 cm (Stace, 2010) with lower species diversity than restored bog areas. They featured eroded gullies, without gully blocking as was the case for “restored bog,” therefore drier with water run‐off. No predator control was practiced, and there were some sheep.

**FIGURE 3:**
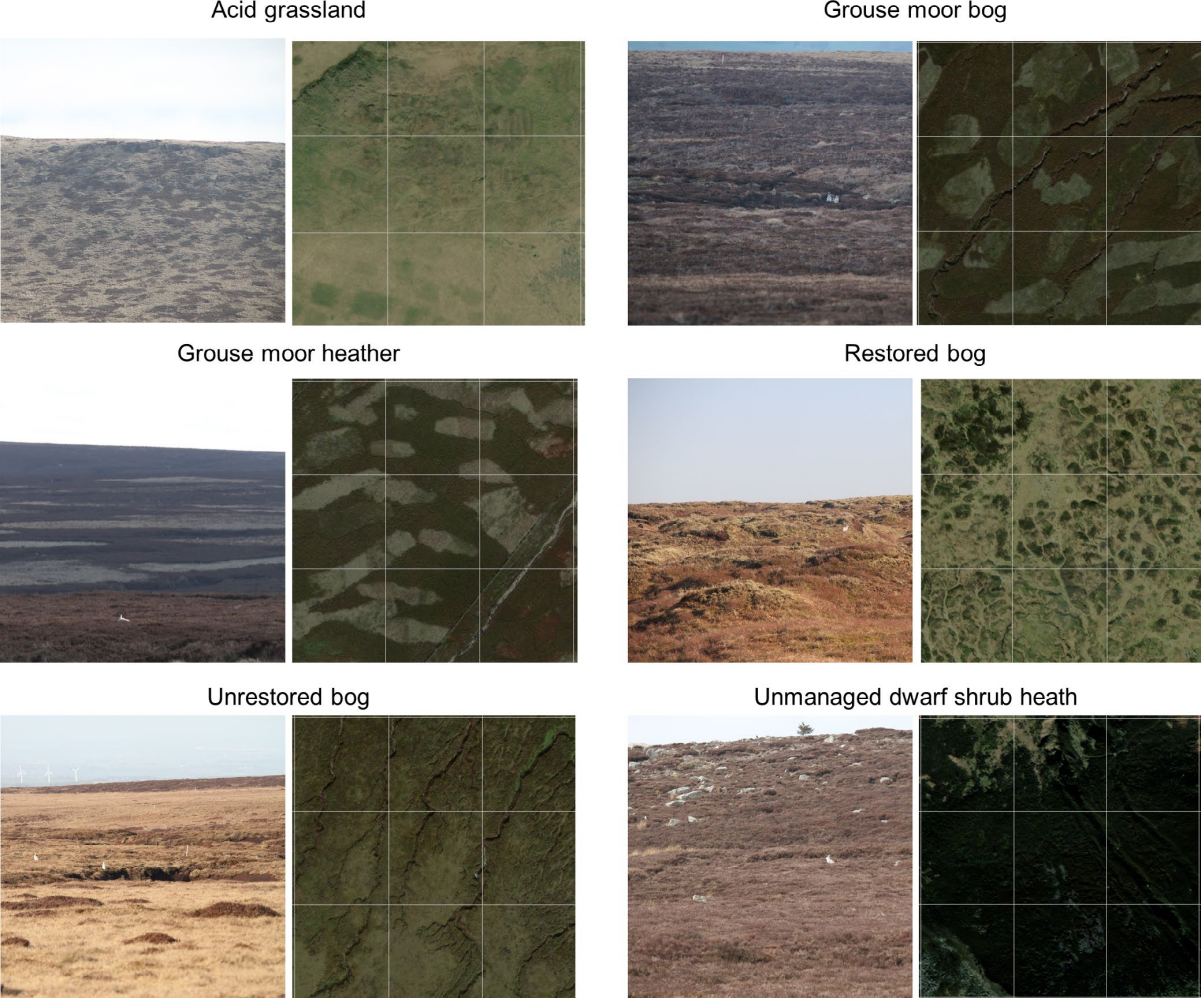
Photographs of each of the habitat classes. For each habitat class, the left field photograph is taken from the ground. The right side photographs are aerial images at 300 m by 300 m with a 100 m fishnet grid overlain, for scale. Source: ArcGIS ESRI "WorldImagery" downloaded 3 August 2021. Colors are natural, not enhanced. Note each field photograph also contains an example mountain hare observation

Ground and aerial photographs showing habitat classes appear in Figure 3. Table 1 lists vegetation communities. Habitat class data for hare observations, transect lengths, and surveyed area size were then determined using “extract” function in package Raster (Hijmans & van Etten, 2012) within R (R Core Team, 2021).

**TABLE 1:** Ecosystems and habitat classes used in this research and the plant communities within these areas, as described by the British National Vegetation Classification (NVC) (Elkington et al., 2001; Hall et al., 2004; Jackson, 2000; JNCC, 2015; Natural England, 2005; Rowland et al., 2017)

Ecosystem	Habitat class	NVC category
Blanket bog	Restored bog	M1 and M2 *Sphagnum* bog pools M3 and M20 *Eriophorum* bog pools M4 Carex rostrata—*Sphagnum recurvum* mire M5 Carex rostrata—*Sphagnum squarrosum* mire M6 *Carex—Sphagnum* mires M9 Carex rostrata—*Calliergon cuspidatum*/*giganteum* mire M15 *Scirpus cespitosus—Erica tetralix* wet heath M16 *Erica tetralix—Sphagnum compactum* wet heath M19 *Calluna—Eriophorum* blanket mires
	Unrestored bog	As for restored bog
	Grouse moor bog	As for restored bog
Upland dry heath	Grouse moor heather	H1 *Calluna—Festuca* heath H8 *Calluna—Ulex* heath H9 *Calluna—Deschampsia* heath H10 *Calluna—Erica* heath H12 *Calluna—Vaccinium* heath H18 *Vaccinium—Deschampsia* heath M19 *Calluna—Eriophorum* blanket mires
	Unmanaged dwarf shrub heath	H1 *Calluna—Festuca* heath H8 *Calluna—Ulex* heath H9 *Calluna—Deschampsia* heath H10 *Calluna—Erica* heath H12 *Calluna—Vaccinium* heath H18 *Vaccinium—Deschampsia* heaths
Acid grassland	Acid grassland	U1 *Festuca ovina—Agrostis capillari* ‐ *Rumex acetosella* grassland U2 *Deschampsia flexuosa* grassland U4 *Festuca ovina—Agrostis capillaris—Galium saxatile* grassland U5 *Nardus stricta—Galium saxatile* grassland U6 *Juncus squarrosus—Festuca ovina* grassland *W16 Quercus* spp.*—Betula* spp.*—Deschampsia flexuosa* woodland (for bracken)

### 2.3 Surveys

When planning surveys, we perceived a random stratified approach (Morrison et al., 2010) might miss local concentrations of mountain hares (Flux, 1962) with typical small home ranges from ~10 ha (Rao et al., 2003) to ~100 ha (Hewson & Hinge, 1990). We, therefore, designated survey sites as 5 × 5 km, potentially identifying hare density patterns over a few hundred meters and large enough to encompass all habitat classes. During pilot surveys, we observed mountain hares up to 700 m range and so conducted transects in sampled 1 km squares of the Ordnance Survey grid (OS Explorer Map^1^, ^2015^), achieving continuous coverage probability >.01. The perimeter of each square was surveyed as a circuit, walking all four sides as one continual transect. By walking all cardinal directions, we intended this to account for sampling differences arising from slope, weather, or lighting. We considered each 1‐km transect to be independent. At adjoining corners of squares, there was overlap of visual coverage (at a subsequently modeled range 520 m), meaning corners were surveyed twice a year compared with remaining areas surveyed once. We assessed this coverage (Table A1) using Pearson’s chi‐square test, which reported no significant difference in proportion of habitat classes surveyed twice, versus once: χ^2^ (5) = 3.588, *p* = .61. Hence, we did not modify estimates for differing coverage probabilities. Therefore, we met standard distance sampling assumptions with survey effort acting as denominator for encounter rate (Buckland et al., 2001, 233–235; Buckland et al., 2004, 224; Buckland et al., 2015, 27).

To meet our aim of surveying the entire mountain hare population at our sites, our study sampling was designed to make efficient use of limited staff time and good weather days. From BRC records, we noted 37% of historic observations were on 23% of the study area: Bleaklow and Margery Hill, with 4 km of non‐surveyed land between them (Figure 1). Thus, we configured the 5 × 5 km sites atop these two hills, acknowledging that ensuing density estimates might be higher than elsewhere in the wider park. We surveyed alternate 1 km^2^ squares, that is, 13 squares on Bleaklow, 13 squares on Margery Hill. Surveys commenced for 2017, repeated in 2018, 2019, 2020, and 2021 with the same 26 squares being surveyed each year (Figure 1).

We added an additional 5 × 5 km site on Holme Moss with 13 more squares in 2018, repeated in 2019. During 2019, we extended surveys to achieve an estimate for the entire Peak District. Because the remaining park was much larger, for logistical reasons we configured remaining surveys of areas as 26 random 1 km^2^ squares (“peripheral areas”), with 6 squares deliberately chosen as pairs, for efficiency (Figure 1).

Survey transects followed each 1 km^2^ square perimeter, guided by GPS (Garmin 64MapST, 15m accuracy), and were conducted January through April. The survey schedule randomized squares the first year, maintaining the same schedule each year as logistics allowed. Each side of the square was surveyed once, looking on both sides of the transect, walking very slowly, and taking 2–5 h. Surveyors scanned ahead with binoculars every 200 m to locate hares or groups of hares in the undulating terrain. Only observations made while walking along the transect line were included in the analysis.

Mountain hare observations were made using standard distance sampling methods, recording date, time, grid reference, cluster size, distance to hare (Nikon ProStaff7i laser range finder, accuracy 1m), and angle (compass and angle board) (Buckland et al., 2001). Potential double counts for observation were discounted. Previous studies described difficulties of daytime surveys for mountain hares, as this nocturnal species often hides by day, revealing itself by flushing from cover, a difficulty associated with tall heather on grouse moor habitats, contributing to imprecise density estimates (Bedson, Thomas, et al., 2021; Newey et al., 2003, 2018) To evaluate whether this behavior affected the detection process, we categorized hare activity upon first being observed, as stationary (lying or sat up); moving (walking, running, or feeding); or flushing (emerging from cover). Surveys were conducted under similar conditions for comparable previous studies in clear weather with wind speed <20 mph (e.g., Newey et al., 2018). We assumed stronger winds did not influence hare detections (e.g., Flux, 1962), but caused difficulties holding the laser range finder steady. No surveys were conducted with snow present.

### 2.4 Distance modeling

For Bleaklow and Margery Hill, mountain hare observations were attributed to the habitat class on which the animal was first seen (as represented in Figure 1). To consider the possibility of field measurement errors (GPS, laser range finder, and angle board) affecting habitat class assignment, within ArcGIS we applied buffers of 25 m circles to all observations and found 97.3% of these lay wholly within the observation’s extracted habitat class; 2.7% straddled two habitat classes. We accepted this as tolerable systematic error. We excluded Holme Moss and peripheral areas from habitat analyses as they were not surveyed every year, retaining them for discrete “area only” estimations.

**FIGURE 4:**
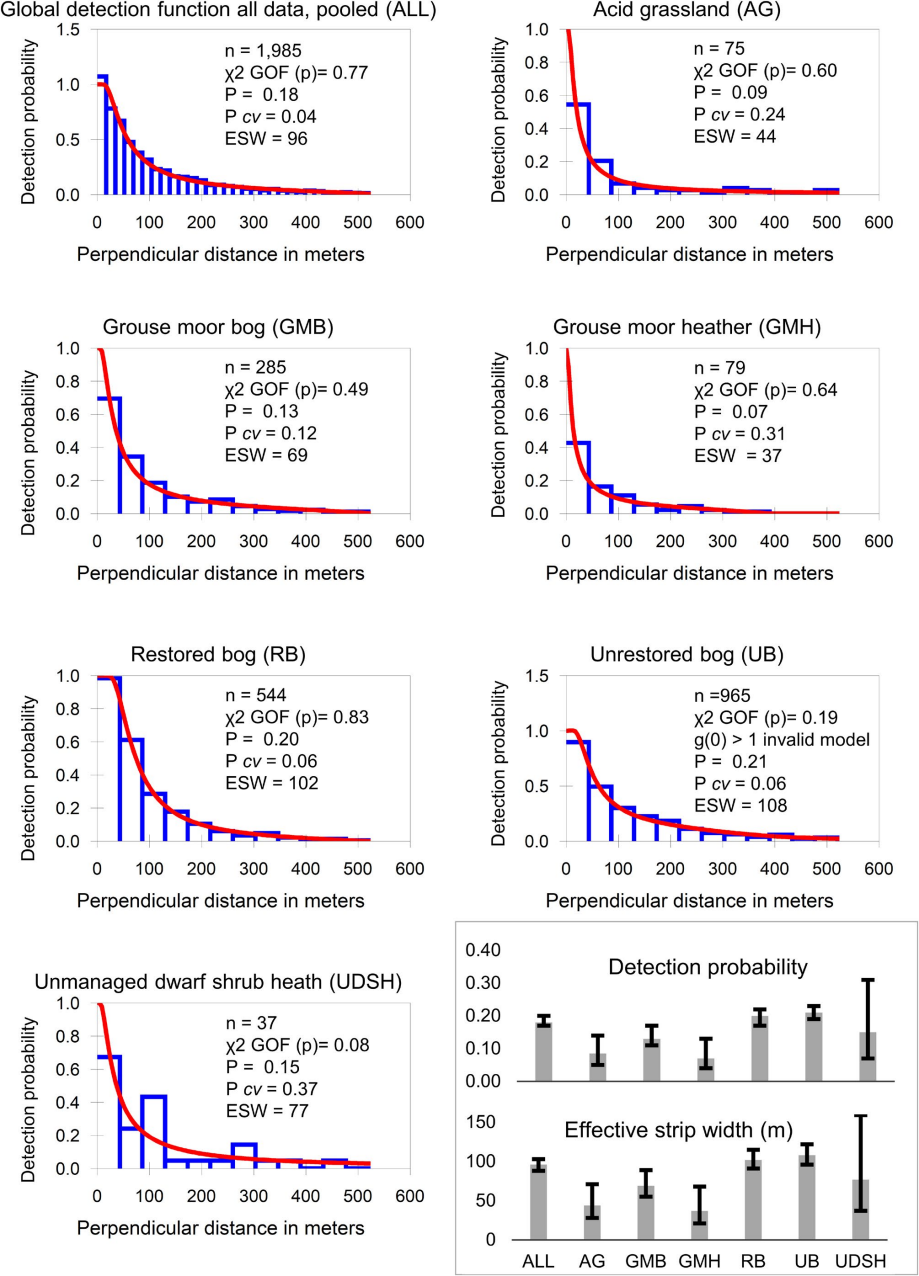
Histograms for Bleaklow and Margery Hill distance sampling data from 2017 to 2021 (1985 observations) fitted with the hazard‐rate model truncated at 520 m. The first histogram shows all data, pooled, as used for reporting. The subsequent six histograms show detection functions when stratified by habitat class with parameters: *n* = sample size; χ^2^ GOF (*p*) = chi‐square goodness of fit *p*‐value; *P* = detection probability *P cv* = coefficient of variation; ESW = effective strip width in meters. Detection function for unrestored bog reports detection probability at the transect line >1, that is, invalid model. The column charts bottom right show detection probability and effective strip width estimates with 95% confidence intervals. All = from global detection function all data, pooled, showing much narrower confidence intervals than the subsequent six columns where the detection function is stratified by habitat class

We analyzed our data with DISTANCE v.7.3 (Thomas et al., 2010), using different data filtering and model selections. We assessed different truncation distances and bin widths. We compared detection models with three key functions: uniform, half‐normal, and hazard rate, with cosine or polynomial expansion terms (Buckland et al., 2001, 47; Williams & Thomas, 2007). We assessed the suitability of assumptions and models using histograms, quantile‐quantile plots, *χ* ^2^ goodness of fit statistics, and the fit of the detection function close to the transect line g(0). We compared and sought simple models with few parameters, lower AIC values between models using the same data selection, higher *χ* ^2^ goodness of fit statistics, and lower detection probability *cv* values (Buckland et al., 2001). The furthest observation distance was 780 m. We truncated the data at a range of 520 m. The hazard‐rate model provided its characteristic wide shoulder and steep drop off of the detection function with increasing perpendicular distance. With data truncated at 520 m, this provided a high *χ* ^2^ goodness of fit statistic (0.77) for the detection function, with *p* = .18 and low detection probability *cv* = 0.04 (Table A2, Figure 4). Both the uniform and half‐normal models failed to achieve a suitable (i.e., >0.05) *χ* ^2^ goodness of fit statistic with most data selections.

We compared two approaches to stratification by habitat: (1) global detection function using pooled data, this required three parameters, reporting AIC 22,148.43, global *P cv* = .04; (2) strata (i.e., habitat class)‐specific detection function, this required 16 parameters, reporting AIC = 22,110.73, ∆AIC = 37.70 (Figure 4). The lower AIC of the strata‐specific detection function indicated this model was best. However, for some habitats this estimated high *P cv* values: acid grassland = .24; grouse moor heather = .30; and unmanaged dwarf shrub heath = .36, leading to greater uncertainty for density estimates. Additionally, the detection function for unrestored bog was invalid (*g*(0) > 1) and the sample size for unmanaged dwarf shrub heath was 37 observations, below that recommended by Buckland et al. (2001), exacerbating doubts about estimate validity. Meanwhile, attempts to use strata as covariates resulted in greater AIC values and were dismissed.

We considered how the strata‐specific detection function varied by habitat class. Outlying hare observations were achieved at long ranges: acid grassland 747 m; grouse moor bog 623 m; grouse moor heather 565 m; restored bog 780 m; unrestored bog 732 m; and unmanaged dwarf shrub heath 566 m. Figure 3 shows example long‐range detections. Effective strip widths varied considerably: acid grassland 44 m; grouse moor bog 69 m; grouse moor heather 37 m; restored bog 102 m; unrestored bog 108 m; and unmanaged dwarf shrub heath 77 m.

**FIGURE A1:**
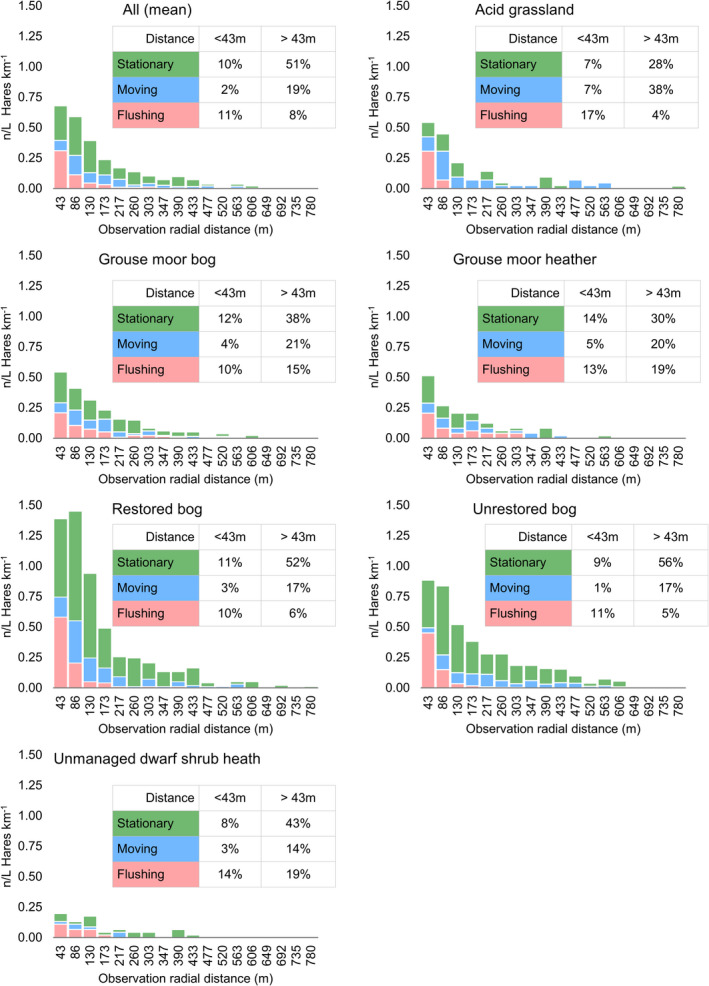
Encounter rate (hares km^−1^) by habitat class, by activity first observed for all Bleaklow and Margery Hill detections *n* = 1999. If groups, activity recorded as that of majority of hares. Histogram distance bin widths arranged at ~43 m increments as Figure 4. Note *x*‐axis = radial distance observer to object, whereas Figure 4 *x*‐axis represents perpendicular distance, hence differences between the two charts. When comparing summed encounters occurring either within or beyond 43 m for each habitat class, the highest proportion of activity was 56% of hares on unrestored bog beyond 43 m as stationary. Proportionately nearly twice as many observations on grouse moor bog or heather were of flushing hares, compared with restored or unrestored bog. Chart excludes records of 11 hare encounters where activity went unrecorded: 3 on restored bog and 8 on unrestored bog

We assessed whether hare detectability (hiding behavior) varied between habitat classes. For all observations, hare activity was recorded as 61% stationary, 21% moving, and 19% flushing from cover, varying per habitat class and radial distance. To test for relationships between these factors, we calculated encounter rate, allocating untruncated observations to 18 radial distance bin widths of ~43 m (Figure A1), that is, resembling habitat stratified detection function histograms (Figure 4). Some activities were not observed at certain ranges, so log‐linear analysis was not possible (Field et al., 2012, 837). Therefore, we grouped observations as within or beyond 43 m, evaluating with Pearson's chi‐square test (multiplying to encounters per 100 km). This showed significant association of activity, habitat class, and observation distance χ^2^ (16) = 224.76, *p* < .001. Hares were more likely to flush on unmanaged dwarf shrub heather (33% of observations) grouse moor heather (32%), grouse moor bog (25%), than on acid grassland (21%), restored bog (16%), and unrestored bog (16%). On unmanaged dwarf shrub heath and grouse moor heather, proportionally more hares flushed at greater distances. However, absolute encounter rates (hares km^−1)^ of flushing hares were as follows: acid grassland 0.38, grouse moor bog 0.54, grouse moor heather 0.51, restored bog 0.91, unrestored bog 0.67, and unmanaged dwarf shrub heath 0.26. These findings did not support the hypothesis that more hares might be lying undetected, that is, perhaps hiding and not flushing, on grouse moor areas. For all the above reasons, we did not believe a stratified detection function would be more informative. Therefore, we used the same global detection function using pooled data for all stratification queries, with only encounter rate and cluster size varying by strata per habitat class and or year (Buckland et al., 2001, 89–91).

We stratified the sampling data and reported in four ways: (1) by habitat class, that is, pooling all observations in each habitat class over the 5 years together; (2) by year, that is, pooling all observations in each year, without habitat information; (3) by habitat and by each year, that is, 6 habitats × 5 years = 30 strata; and (4) by area only and to enable the 2019 population estimate. This was accomplished within software distance, using the same data, each time allocating transects and observations to different strata definitions (Thomas et al., 2010). Estimates for the survey year 2019 also used data truncated at 520 m and the hazard‐rate model; inevitably its global detection function *f*(0) differed slightly from the smaller data set of Bleaklow and Margery Hill. We reported parameters and estimates with 95% confidence intervals. Comparisons between strata used the *t*‐statistic based on the Satterthwaite approximation, accounting for unequal sample sizes (Buckland et al., 2001, 84–86). This test takes into account the lack of independence of data arising from using a common detection function between strata. We evaluated significance with a Bonferroni‐corrected *p*‐value and also calculated effect sizes (Field et al., 2012).

Abundance for the Peak District National Park was calculated for 2019 based on the additional survey effort. The 2019 survey showed very strong density fall off from center to edge of the Park. Therefore, to determine the extent for calculating abundance, we created an alpha hull shape measuring 325 km^2^, from BRC hare records (Figure 1). We discarded six outlying records to cover only the known range of hares. This alpha hull shape differed very slightly from our survey area, so we merged them based on habitat classes to total 358 km^2^. Abundance was calculated for each of Bleaklow and Margery Hill and Holme Moss and peripheral areas, multiplying density estimates by area.

## 3 RESULTS

### 3.1 Observations

**FIGURE 5:**
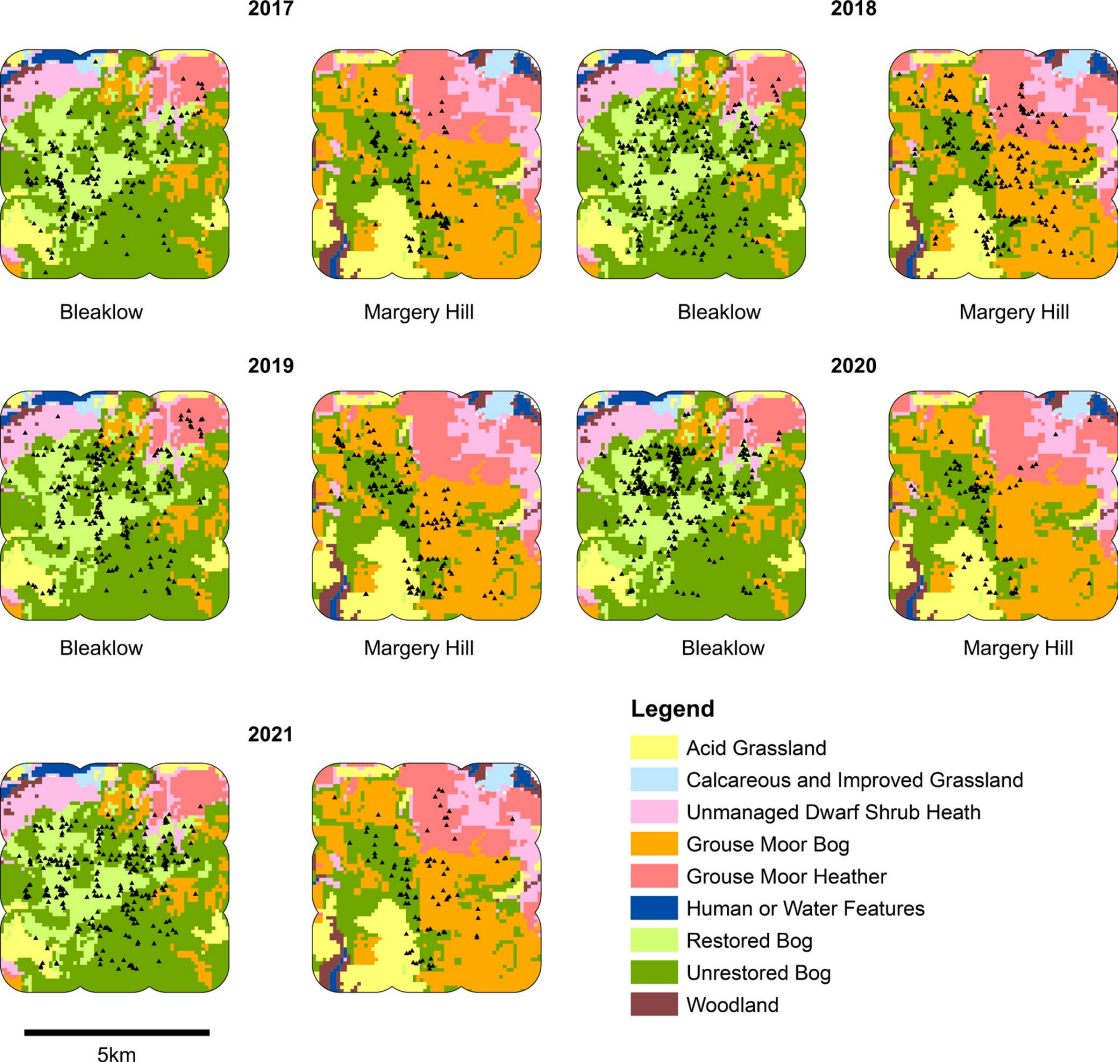
Distance sampling observations for Bleaklow and Margery Hill survey sites with 800 m buffer, for years 2017 to 2021. Habitat classes as legend. Bleaklow map origin is British National Grid Reference SK Easting 308000 Northing 394000. Margery Hill survey site is duly positioned 4 km to east. Black triangles indicate all observed mountain hares (untruncated data)

**FIGURE A2:**
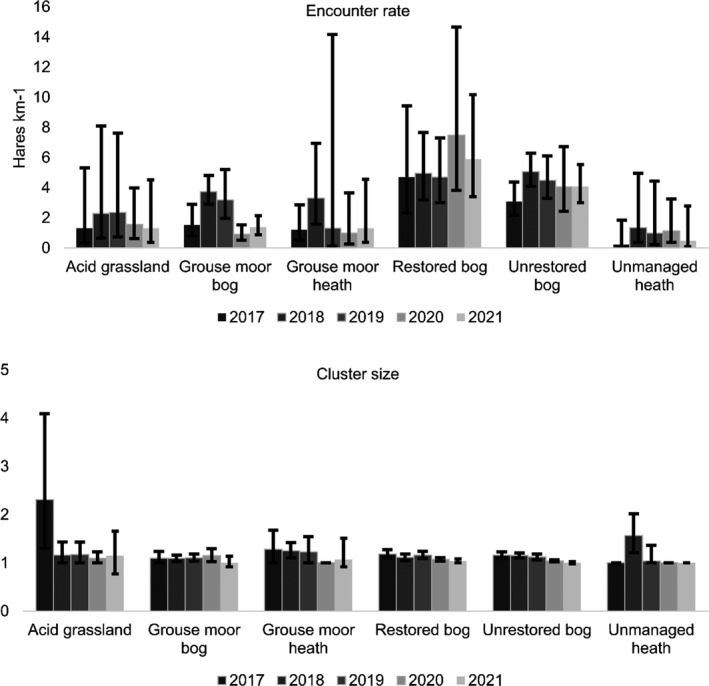
Encounter rate (mountain hares km^−1^) and cluster size estimates for each habitat class and year based on Bleaklow and Margery Hill data. In total the number of hares recorded (before data truncation) was 385 in 2017; 622 in 2018; 517 in 2019; 434 2020; 458 in 2021

**FIGURE A3:**
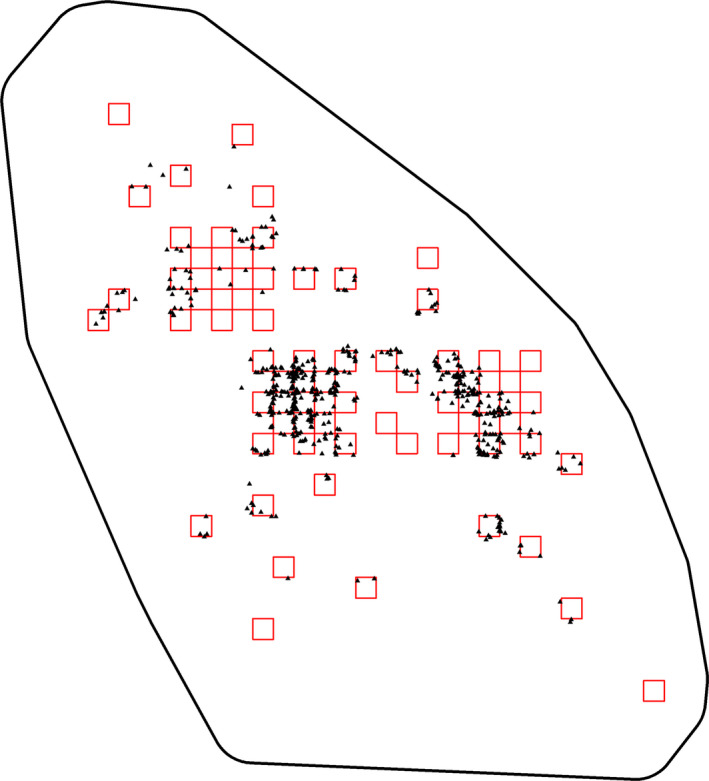
Distance sampling observations for the entire Peak District survey for 2019. Map origin is British National Grid Reference SK Easting 390000 Northing 370000. Transects are red 1 km squares. Black triangles indicate all observed mountain hares (untruncated data)

In 2017, Bleaklow and Margery Hill surveys covered 121 km of transects, recording 304 detections; 2018 covered 122 km with 504 detections; 2019 covered 113 km with 401 detections; 2020 covered 123 km with 402 detections; and 2021 covered 121 km with 374 detections (Table 2; Figure 5). Encounter rate estimates varied from highest 7.5 (95% CI: 3.8–14.7) mountain hares km^−1^ on restored bog in 2020 to lowest 0.2 (95% CI: 0.0–1.8) mountain hares km^−1^ on unmanaged dwarf shrub heath 2017 (Table A3, Figure A2). Cluster sizes were slightly above 1.0; most encounters were single hares (Table 2, Table A3, Figure A2). The surveys of 2018 on Holme Moss covered 60 km with 89 observations and 2019 covered 58 km with 50 observations. Peripheral areas in 2019 covered 113 km with 101 observations (Table 2, Figure A3).

**TABLE 2:** Stratified distance sampling survey parameter estimates. Data selection based on 520 m truncation with hazard rate model

	*n*	*L*	*n*/*L*	*n*/*L cv*	*n*/*L* LCL	*n*/*L* UCL	*K*	*E* (s)	*E* (s) *cv*	$\hat{D}$	$\hat{D}$ *cv*	$\hat{D}$ LCL	$\hat{D}$ UCL
Habitats													
Acid grassland	75	42.3	1.8	0.23	1.1	2.8	36	1.28	0.05	11.8	0.24	7.3	19.2
Grouse moor bog	285	133.9	2.1	0.12	1.7	2.7	85	1.09	0.01	12.2	0.13	9.4	15.8
Grouse moor heath	79	48.6	1.6	0.23	1.0	2.6	23	1.18	0.03	10.0	0.24	6.1	16.6
Restored bog	544	97.8	5.6	0.12	4.4	7.1	54	1.12	0.01	32.6	0.12	25.2	42.2
Unrestored bog	965	233.0	4.1	0.07	2.6	4.8	117	1.12	0.01	24.4	0.08	20.6	29.0
Unmanaged heath	37	45.3	0.8	0.30	0.4	1.5	47	1.12	0.04	4.8	0.30	2.6	8.8
Years													
2017	304	120.9	2.5	0.20	1.7	3.8	26	1.18	0.02	15.5	0.21	10.1	23.9
2018	504	121.6	4.1	0.10	3.3	5.2	26	1.14	0.01	24.7	0.11	19.6	31.5
2019	401	112.5	3.6	0.14	2.6	4.8	26	1.13	0.01	21.1	0.15	15.4	29.0
2020	402	123.1	3.3	0.25	1.9	5.5	26	1.05	0.01	17.9	0.26	10.6	30.8
2021	374	120.8	3.1	0.18	2.1	4.4	26	1.13	0.01	18.3	0.18	12.6	26.8
Survey areas 2019													
Bleaklow	246	56.7	4.3	0.14	3.2	5.9	13	1.09	0.01	27.2	0.15	19.9	37.9
Margery Hill	155	55.8	2.8	0.30	1.5	5.3	13	1.16	0.03	18.6	0.30	9.7	35.5
Holme Moss	50	57.5	0.9	0.26	0.5	1.5	13	1.22	0.03	6.1	0.27	3.4	10.9
Peripheral Squares	101	113.0	0.9	0.19	0.6	1.3	26	1.19	0.04	6.2	0.20	4.1	9.4

Note

*n* = encounters; *L* = line length km; *K* = number of transects; *E*(s) = mean cluster size; $\hat{D}$ = density estimate km^−2^; *cv* = parameter coefficient of variation; LCL & UCL = 95% confidence intervals. $\hat{D}$ is calculated with probability density function *f*(0) and *f*(0) *cv*. (Buckland et al., 2001, 84,85). “Habitats” data source is Bleaklow and Margery Hill only with probability density function *f*(0) = 0.010467 and *f*(0) *cv* = 0.0407 and represents 2017 to 2021 totalled effort and encounters, mean cluster size and density estimate values. “Years” data source: Pooled by year (not by habitat) for Bleaklow and Margery Hill only with same probability density function. Survey areas 2019 is modelled with all data for all areas for all areas (2225 observations) with probability density function *f*(0) = 0.011522 *f*(0) *cv* = 0.0407. However the table just reports estimates for the surveyed areas for 2019 only.

### 3.2 Density and abundance

**FIGURE 6:**
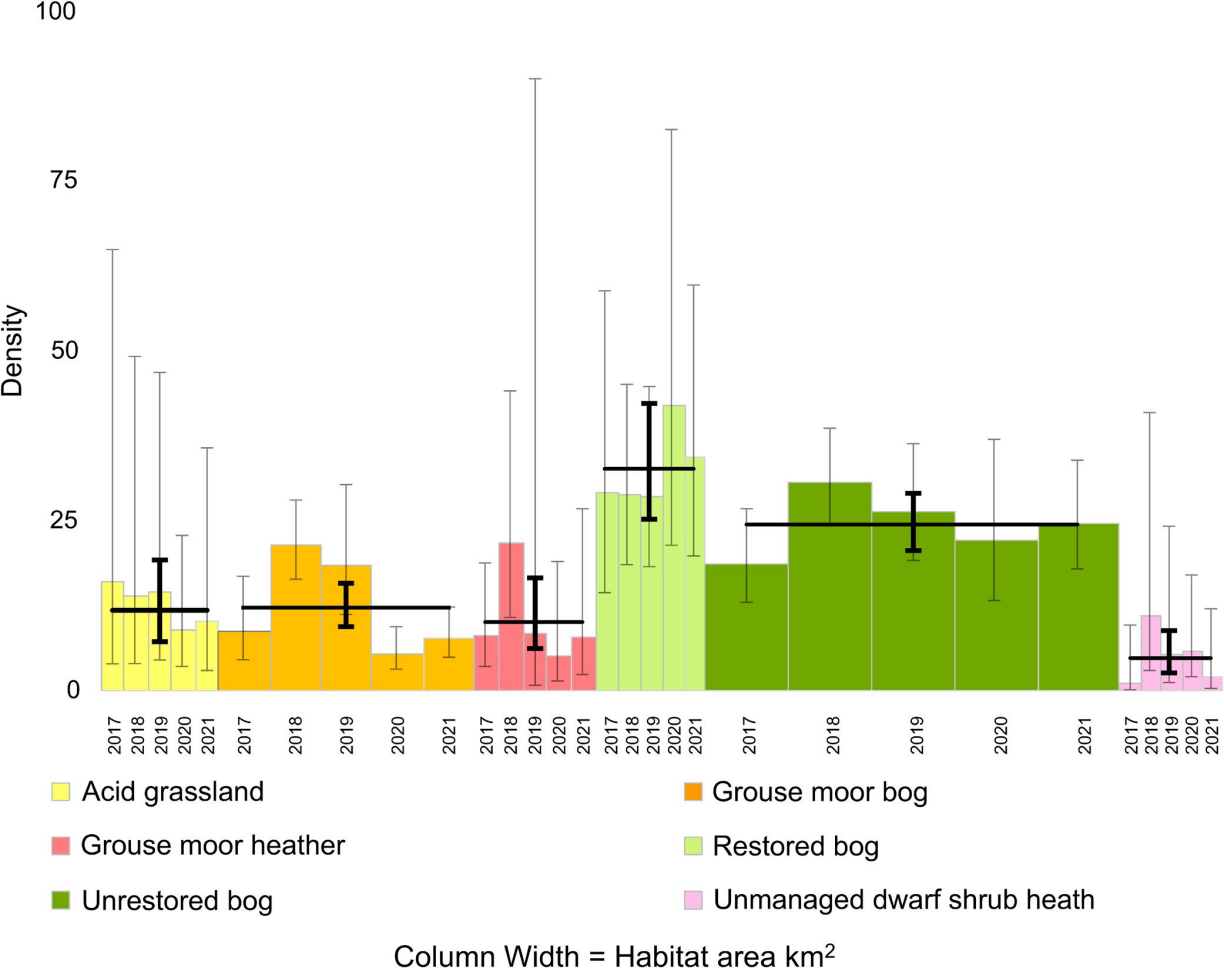
Estimates of abundance of mountain hares by habitat class, each year, as reported by distance sampling analysis for Bleaklow and Margery Hill only. *x*‐axis column widths represent habitat area in km^2^ which were as follows: acid grassland 8.5; grouse moor bog 18.4; grouse moor heather 18.7; restored bog 7.9; unrestored bog 29.8; and unmanaged dwarf shrub heath 7.4. Column height is mean density estimate (D). Column error bars indicate lower and upper 95% confidence limits on D. The shaded column area, therefore, represents the abundance of hares on each habitat type each year based on point density values. Black horizontal bars indicate mean density value for each habitat over the 5 years, with black vertical error bars showing 95% confidence limits (following Clymo, 2014, 230)

On Bleaklow and Margery Hill, the 5‐year mountain hare point estimates of density hares km^−2^ per habitat class were restored bog = 32.6 (95% CI: 25.2–42.2), unrestored bog = 24.4 (20.6–29.0), grouse moor bog = 12.2 (9.4–15.8), acid grassland = 11.8 (7.3–19.2), grouse moor heather = 10.0 (6.1–16.6), and unmanaged dwarf shrub heath = 4.8 (2.6–8.8) (Table 2, Figures 5 and 6). There were significant differences for 10 paired comparisons of habitats (Table A4). Hare densities on restored bog were significantly higher (*p* < .05) than all other habitats except unrestored bog; densities in the former were 34% higher than the latter: *t*(1.92) = 99.03, *p* = .057, *r* = .19. Unrestored bog also showed significantly higher densities than the other classes. Acid grassland, grouse moor heather, and grouse moor bog were similar. Grouse moor bog hare density was not significantly higher than grouse moor heather *t*(0.76) = 47.19, *p* = .449, *r* = .11. Acid grassland and grouse moor bog were significantly higher than unmanaged dwarf shrub heath. Grouse moor heather was higher than unmanaged dwarf shrub heath *t*(1.90) = 43.10, *p* = .064, *r* = .28. When comparing habitats within each individual year, many of these differences were often apparent in individual years (Tables A3 and A5).

From 2017 to 2018, unrestored bog showed a significant increase in hare density from 18.7 hares km^−2^ (95% CI: 13.0 to 26.7) to 30.5 hares km^−2^ (95% CI: 24.3–38.6), *t*(2.64) = 57.93, *p* = .011, *r* = .33 (Tables A3 and A4). From 2017, grouse moor bog reported hare density increasing significantly from 8.7 hares km^−2^ (95% CI: 4.5–16.8) to 21.4 hares km^−2^ (95% CI: 16.4–28.0), *t*(3.29) = 40.07, *p* = .002, *r* = .46. On grouse moor bog, hare density from 2019 to 2020 decreased significantly from 18.3 km^−2^ (95% CI: 11.2–30.3) to 5.5 km^−2^ (95% CI: 3.2–9.4), *t*(2.88) = 21.65, *p* = .009, *r* = .53 and this was the only significant decrease in any habitat type between years.

On Bleaklow and Margery Hill, annual density estimates showed a significant increase by 59% from 2017 to 2018 from 15.5 hares km^−2^ (95% CI: 10.1–23.9) to 24.7 hares km^−2^ (95% CI: 19.6–31.5), *t*(2.29) = 57.56, *p* = .025, *r* = .29) (Table 2). Density then dropped 15% to 21.1 hares km^−2^ (95% CI: 15.4–29.0) in 2019 and by 15% to 17.9 hares km^−2^ (95% CI: 10.6–30.8) in 2020. From 2020 to 2021, density reported an increase by 2% to 18.3 hares km^−2^ (95% CI: 12.6–26.8).

**FIGURE A4:**
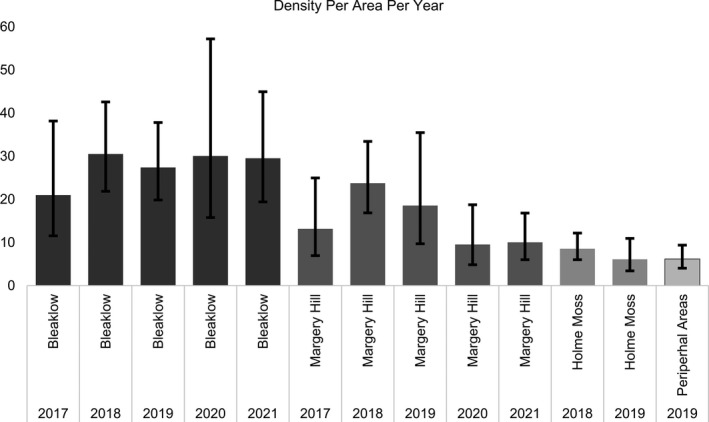
Estimates of total mountain hare density per year per survey area, based on pooled observations each year. Error bars indicate 95% lower and upper confidence limits. Upon Bleaklow and Margery Hill there was a 59% increase in density from 2017 to 2018. This was followed by a 15% decrease 2018–19%; 15% decrease 2019–20; 2% increase 2020–21. Density upon Bleaklow and Margery Hill was significantly higher than Holme Moss when it was surveyed in 2018 and 2019 and also significantly higher than peripheral areas in 2019

Of the 2019 survey areas, the highest density of hares was reported for Bleaklow with 27.2 hares km^−2^ (95% CI: 19.9–37.9), significantly higher than any other area (Tables 2 and A4, Figure A4). Margery Hill density was also high at 18.6 hares km^−2^ (95% CI: 9.7–35.5). Holme Moss had low density of mountain hares 6.1 hares km^−2^ (95% CI: 3.4–10.9), and this was similar to the peripheral areas 6.2 hares km^−2^ (95% CI: 4.1–9.4).

**FIGURE A5:**
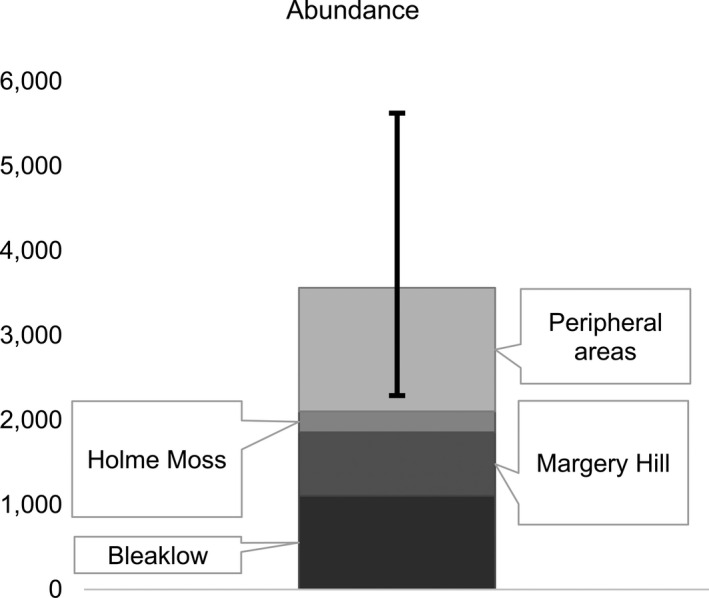
Abundance estimate for Peak District for year 2019, based on density estimates derived from pooled observations for the four different survey areas indicated by callouts. Error bars indicated 95% lower and upper confidence limits for total abundance. Source data from Table A6

For 2019, abundance for the Peak District study area (alpha hull shape + surveyed areas) estimated 3562 hares (95% CI 2291–5624) (Table A6; Figure A5). Bleaklow was 11% of area and accounted for 31% of hares; Margery Hill was 11% of area and 21% of hares; Holme Moss 11% area and 7% of hares; and peripheral areas 66% area and 41% of hares.

## 4 DISCUSSION

We report strong evidence that mountain hare density differs between peatland habitat types. We found intensely localized hare abundance, which we attribute to characteristics of the habitat classes. There appears a clear association between restored bog habitat and high mountain hare densities. Many studies of peatland restoration describe levels of degradation and potential effects of recovery interventions upon hydrology, water tables, soil quality, carbon and methane storage, and vegetation (Alderson et al., 2019; Bain et al., 2011; Holden et al., 2007; Page & Baird, 2016). Few studies show how vertebrates, particularly mammals, may benefit from peatland improvement (Andersen et al., 2017; Littlewood et al., 2021). Our research suggests that restored bogs can have a measurable conservation impact on vertebrate populations. This is encouraging, because many sensitive ecosystems are in such poor condition and resources for restoration are limited (Andersen et al., 2017).

For the United Kingdom, this study represents the first such density estimate comparison based on surveys of live mountain hares (i.e., not game bags), using geospatial measurements of animal occurrence and comparing densities across the full range of habitat classes used by this species. Our findings complement other research in Europe that describe mountain hare habitat utilization: preferences for thickets of *Salix*, *Betula*, and *Picea* with dense understorey in Scandinavian woodland (Hiltunen et al., 2004); preference for dwarf mountain‐pine (*Pinus mungo*) regardless of patch size in the Alps (Bisi et al., 2013); and preference for moorland over woodland in Scotland (Rao et al., 2003). The mountain hare densities we recorded are higher than many comparable populations in Europe. Notable high densities elsewhere include populations on heather moorland in Scotland (Watson & Hewson, 1973 ~280 km^−2^; Watson et al., 1973 ~200 km^−2^; Newey et al., 2018 ~200 km^−2^) and on predator‐free heather dominated islands off mainland Sweden (~400 km^−2^, Angerbjorn & Flux, 1995). Separately, snowshoe hare (*L*. *americanus*) densities reach up to 300 km^−2^ in boreal forests (Krebs et al., 2001).

### 4.1 Degraded habitats

We observed wide variation of hare density between habitat types. We found significant differences between habitat classes, which imply contrasts in vegetation diversity, forage quality, or attractiveness to hares. We detected a significant increase in density between 2017 and 18 followed by 2–3 years of decrease.

The Bleaklow surveys included 20 1‐km^2^ squares, which up to 2003 comprised eroded bare peat (Proctor et al., 2013) or low levels of co‐dominant heather (Anderson & Yalden, 1981). On those, Yalden (1971) recorded hares in only 8 1‐km squares, and as single hare observations. By contrast, our surveys of 2017–21 in those same areas, now as restored bog, showed high densities of mountain hares, that is, 32.6 (95% CI: 25.2–42.2) hares km^−2^; in 2019 for Bleaklow overall 27.2 (95% CI 19.9–37.9) hares km^−2^. This clearly suggests a positive impact of bog restoration on hare density. These restored areas have been shown to support higher floral diversity (Pilkington et al., 2016), which we suggest is attractive and beneficial to hares. Restoration, lime, and fertilizer applied to bare peat, potentially provided a lingering amount of phosphorous and nitrogen in the vegetation (Alderson et al., 2019), affording nutritional benefits to hares (Hewson, 1989; Miller, 1968; Watson et al., 1973). Such might contribute to animal health and higher numbers (Watson et al., 1973). However, it is not clear whether food availability or nutritional quality limits hare populations (Keith, 1983; Newey et al., 2010) so it is hard to make inferences that food is the main cause of differences in hare density between habitats. It is also conceivable that where restoration elevated the water table this created more water and moisture availability for mountain hares, particularly important during summer. Restored bog areas contained eroded gullies used by mountain hares for shelter and movement pathways. Taking advantage of the intricate micro‐topography, during bad weather, hares could simply move ~20 m to new shelter among peat hags and gullies. The eroded gullies also existed in 1971 (Bower, 1961), and again this implies the restoration efforts themselves contributed to high hare numbers.

Unrestored bog areas also showed consistently high mountain hare encounter rates and density estimates. Density on restored bog was 34% higher than unrestored bog, implying restoration benefits were proving supportive. Unrestored bog areas were similar to restored bog with many eroded gullies. However, unrestored bog areas featured extensive swathes of cotton grass with small pockets of heather; not the diverse micro‐mosaic patchwork of assorted grasses, heather, ferns, and moss species seen on restored bog. Therefore, peat fertilization and diverse vegetation replanting on restored bog may have contributed to higher numbers of hares. The absolute extent of unrestored bog and its high densities made this the most important habitat for sustaining this hare population.

The presence of grouse moors was not associated with the highest mountain hare densities. Grouse moor bog showed significantly lower density than unrestored bog, despite having similar vegetation and with gullies present as potential shelter. Hare density on grouse moor bog was slightly higher than grouse moor heather. Both reported densities similar to acid grassland, noted as ecologically impoverished (Anderson & Yalden, 1981). Density on grouse moor bog was significantly higher than unmanaged dwarf shrub heath. Density on grouse moor heather was also notably higher than unmanaged dwarf shrub heath, so the benefit to mountain hare density from heather burning and associated management activities described by Hesford et al. (2019) seemed apparent, as previously reported (Yalden, 1971). Yet, we observed the lower slopes of grouse moor heather were often dry, as also reported by Holden et al. (2015). Frequent extensive heather burning reduced vegetation diversity and cover (Bonn et al., 2009, 178). On some of these areas, no hares were seen. On less frequently burned areas, groups of hares were occasionally observed feeding upon pioneer heather (Hewson, 1962, 1989). The grouse moor bog included deeper mature heather, where some hares hid, though finding movement difficult (Hewson, 1989; Stoddart & Hewson, 1984). Indeed, Yalden (1971) recorded fewer hares in areas of pure *Calluna*. We were unable to ascertain whether predator control on grouse moors was reducing levels of predation and contributing to higher densities of hares.

We estimated lower mountain hare densities on grouse moors than reported in Scotland (Hesford et al., 2019; Newey et al., 2003, 2018). In Scotland, high densities of mountain hares on grouse moors were first reported in four studies. Hewson (1965) reported game bags of 43–295 hares, annually 1955–63 on a 2 km^2^ area. Watson et al. (1973) produced raw count data estimating up to 300 hares km^−2^. Stoddart and Hewson (1984) suggested an association of hares with grouse moors from game bags, estimating hares 42 km^−2^. Watson and Hewson (1973) reported count data, comparing density by habitat, with high densities in valleys 26.3 hares km^−2^, on grouse moors in the Cairngorms 32.6 hares km^−2^; lower at arctic‐alpine areas 7.9 km^−2^, suggesting grouse moor as optimum habitat. More recently, studies in Scotland have shown the persistence of mountain hares measured in terms of occupied range and count indices as associated with moors managed for driven grouse shooting (Hesford et al., 2019, 2020). Very high densities (18–249 hares km^−2^) were recorded on grouse moors in northeast Scotland (Newey et al., 2018). In the Peak District, Yalden (1971, 1984) and Wheeler (2002) found highest counts on heather moorland, followed by bog and acid grassland.

It was, therefore, unexpected to find lower mountain hare density on grouse moors in the Peak District. Possibly mountain hares had shifted habitat use to high elevations, making for higher densities on the biologically diverse and higher altitude bogs. This could be a response to climate change and the rise in annual average temperatures observed in the Peak District (Caporn & Emmett, 2009, 47) and has been forecast across Europe (Leach et al., 2015). On restored and unrestored bog, patches of heather resource were ample, dispersed amidst a variety of other vegetation species and easy for hares to move around. Grouse moor bog had similar vegetation species to unrestored bog; grouse moor heather was characterized by heather species. Yet on both grouse moor bog and heather, the *Calluna* existed in such large deep expanses that movement for hares could be difficult. It may be that intense heather burning resulted in inferior vegetation quality or diversity compared with Scotland. We speculate that Peak District heather moorland overlays acidic rock, which may contribute to lower forage quality and lower hare densities (Watson et al., 1973). On grouse moor bog, there was a significant increase in mountain hare density 2017–18 and a significant decrease in 2019–20. On grouse moor heather, there were large reductions in mountain hares in 2018–20. These fluctuations contrasted with the other habitat types, though heather was found in all of them. The forces which govern populations ought to have been similar: weather, availability of food resource within each habitat class, disease, and parasites (Newey, Willebrand, et al., 2007), contributing to similar dynamics. We reflect that in Scotland, grouse moor estates have conducted lethal removal of mountain hares (Patton et al., 2010). We then speculate whether the same occurred on grouse moors within the Peak District, causing lower and fluctuating mountain hare densities.

Mountain hare density on acid grassland showed high variation. While containing much *Nardus* and *Molinia* disliked by mountain hares, some areas contained *Calluna* patches, enabling hares to feed, without trapping them within it. Unmanaged dwarf shrub heath areas mostly reported lowest hare densities. Its deep mature woody *Calluna* was frequently impenetrable. These findings are consistent with previous work by Yalden (1971), Watson et al. (1973), Hewson (1989). Acid grassland and unmanaged dwarf shrub areas were mostly at extent edges, possibly experiencing human pressure from higher road densities, walking paths, sheep farms, and settlements.

### 4.2 Survey efficacy

The use of daylight distance sampling for mountain hares has been criticized as hares are nocturnal and rest up, hiding by day, resulting in lower observed encounter rates (Newey et al., 2018). However, our research achieved large sample sizes and encounter rates with narrow confidence intervals, a function of high densities on Bleaklow and Margery Hill, and demonstrating distance sampling by day can be effective. That said, we had deliberately chosen those areas for survey efficacy. By contrast, in mountain hare surveys on the Scottish Lammermuir hills, Pettigrew (2020) recommended 90‐min surveys by dawn light as hares are more active and visible at this time rather than by midday when dormant. However, this suggestion lacked information regarding imperfect detection process or detection probability so is hard to compare; and those surveys occurred on small accessible areas ~26 km^2^ of relatively flat elevation 420–520 m. By contrast, the Peak District required >120 km of transects and featured steep hills elevation 630 m with transect elevation changes >350 m over 1 km. These hills were often fog shrouded early morning, so dawn surveys were not possible. Consequently, Peak District surveys took up the whole day (2–4 h per square, two squares in a day). Bedson, Thomas, et al. (2021) compares nocturnal survey methods for mountain hares, showing daytime surveys as effective.

We also considered differences in detection process between different habitat classes. Our surveys went on straight line transects, following the Jenkins et al. (1963) method of flushing hares from cover and were applied consistently to all habitat classes. Of note, the assessment of hare activity, that is, numbers of flushing hares, did not provide evidence that our surveys were missing hares hiding in deep heather. Indeed, all habitat classes contained winter vegetation up to ~100 cm height. Given that mountain hares can lie themselves down to ~15 cm height, they can hide in any habitat.

When assembling these analyses, we also considered several alternative habitat class definitions, for example, merging restored and unrestored bog; grouse moor bog and grouse moor heather. Such alternatives did not change the substantive findings that bog habitats reported significantly higher density than managed grouse moor or acid grassland habitats. During surveys, when walking from one habitat to another, we typically observed an immediate abrupt change of encounter rates within <200 m.

We acknowledge that mountain hares may move between habitat classes and we did not employ telemetry to measure this. Hewson (1962, 1989) suggested hares would move by dusk to feed on grouse moor pioneer heather patches. We rarely observed such movement. Both the high elevation restored and unrestored bog areas contained some heather resource, obviating the need for a nightly migration. We analyzed habitat classes based on where each hare was first seen. We acknowledge field measurement factors may have contributed to small errors of habitat class allocation. Hare home ranges may be very small ~0.1 km^2^ (Hewson & Hinge, 1990; Rao et al., 2003). Because our visual range exceeded 700 m and the study layout meant transects were 1000 m parallel to each other, we felt that coverage of home ranges was likely to be comprehensive. Our surveys occurred without snow lie present, which might otherwise prompt hares to seek for heather which might better protrude out of the snow. Notwithstanding these challenges, our surveys achieved global detection probability of 18% of hares, that is, seeing nearly 1 in 5 hares to a range of 520 m. We duly consider distance sampling by day as effective across habitats.

### 4.3 Population fluctuations

In the Peak District since 1971, there were four previous reports of mountain hare abundance suggesting a population of up to ~1000 individuals (Mallon, 2001). The distance sampling survey of winter 2001–2002 using different methods to this paper estimated abundance at ~12,000 hares (CI: 7000–20,000) (Mallon, 2001; Mallon et al., 2003; Wheeler, 2002). We retrieved that data and applied the same analyses as for 2017–21. This revised 2002 density estimate to 9.4 hares km^−2^ (95% CI 6.8 to 12.9); abundance for survey extent 3361 (95% CI 2431–4612) individuals. However, we recommend caution with 2002 values as its survey methodology differed from that of 2017–21: that is, different transect shapes, different locations, no use of binoculars, no laser range finder for measuring the distance to object, no GPS measurement of transect length, and all observations recorded as singles, that is, no clusters.

Estimates for 2017 to 2021 reported high densities upon Bleaklow and Margery Hill. We acknowledge that using these two high‐density areas for 2019 surveys (i.e., as 40% of survey areas), may bias the park‐wide estimate upwards. The Peak District mean abundance estimate for 2019 refers to densities from the wider survey and alpha hull shape, reporting as 3562 (95% CI 2291–5624) individuals.

Therefore, estimates for 2002 compared with 2019 appear similar and suggest a stable population. We speculate whether the increase in densities seen on restored bog has been balanced by a decrease in densities in other areas. Otherwise, the length of this study (2017–21) is too short to detect population cycles, which are subject to complex factors (Newey, Willebrand, et al., 2007). Population dynamics for congeneric snowshoe hare suggest annual fluctuations with observed increases by 25%, or decreases by as much as 75%, linked to food supply and predation (Krebs et al., 2001). Cycle periodicity of mountain hares in Scotland has a range of 4–15 years, with amplitude of up to 90% (Newey, Dahl, et al., 2007), 8 years historically for Irish hare (Reid et al., 2021).

We cannot identify explicit causation for the population fluctuations we observed. Winter 2017–18 was exceptionally severe (UK Met Office, 2020), possibly causing additional mortality. Summer 2018 was extremely hot, potentially contributing to difficult breeding conditions arising from dry vegetation and reduced water availability. Under climate change, the range of mountain hares is forecast to move northwards and to higher elevations (Bedson, Devenish, et al., 2021; Leach et al., 2015; Rehnus et al., 2018), which may result in lower abundances.

This Peak District mountain hare population assessment shows how their confinement to the uplands, and sensitivity to different habitats, makes them a useful mammal species for ecosystem monitoring. They provide an understanding of mammalian responses to climate change: a cold‐niche specialist at the periphery of their climatic range (Harris & Yalden, 2008). We suggest both degrading forces and restoration efforts impact upon hare density. There is substantial variation of density between habitat classes, predisposing the population to local extinction events (Patton et al., 2010). Management agendas should consider how future changes to habitat landcover and land use may affect this mountain hare population.

## CONFLICT OF INTEREST

None declared.

## AUTHOR CONTRIBUTIONS

**Carlos P. E. Bedson** contributed to conceptualization (lead); data curation (lead); formal analysis (lead); funding acquisition (lead); investigation (lead); methodology (lead); project administration (lead); validation (lead); visualization (lead); and writing—original draft (lead); and writing—review and editing (lead). **Philip M. Wheeler** contributed to data curation (supporting); validation (equal); and writing—review and editing (supporting). **Neil Reid** contributed to conceptualization (supporting); funding acquisition (supporting); methodology (supporting); validation (supporting); visualization (supporting); writing—original draft (supporting); and writing—review and editing (supporting). **Wilson Edwin Harris** contributed to writing—review and editing (equal). **David Mallon** contributed to conceptualization (supporting); investigation (supporting); validation (supporting); and writing—review and editing (supporting). **Simon Caporn** contributed to validation (supporting) and writing—review and editing (supporting). **Richard Preziosi** contributed to conceptualization (supporting); formal analysis (supporting); funding acquisition (supporting); methodology (supporting); project administration (supporting); resources (lead); software (supporting); supervision (lead); validation (supporting); visualization (supporting); and writing—review and editing (supporting).

## Data Availability

Observation data are available from the corresponding author upon reasonable request.

## Appendix

**TABLE A1:** Coverage of surveyed area arising from square survey design (Figure 1)

Habitat class		Of which		
	Total surveyed area	Area visited once	Area visited twice	Total area visited
Acid grassland	8.5	7.4	1.2	9.6
Grouse moor bog	18.4	11.9	6.5	24.9
Grouse moor heather	8.7	6.8	2.0	10.7
Restored bog	7.9	2.7	5.2	13.1
Unrestored bog	29.8	18.9	10.9	40.6
Unmanaged dwarf shrub heath	7.4	6.4	1.0	8.4

Note

Those areas at adjoining vertices effectively received two visits in each year. Values are km^2^.

**TABLE A2:** Range of candidate models based on all data for Bleaklow and Margery Hill, pooled. (2017, 2018, 2019, 2020, and 2021)

Data selection	*n*	Model (key)	# para	AIC	ΔAIC	χ^2^ GOF (*p*)	*P*	*P cv*
Truncate at 520 m	1985	Uniform + cosine	3	22353.25	204.82	.00	.30	.01
		Uniform + poly	3	22672.06	523.63	.00	.40	.01
		Half‐normal + cosine	3	22260.09	111.66	.00	.25	.02
		Half‐normal + Hermite	1	22619.32	470.89	.00	.35	.01
		Hazard rate + cosine	3	22152.52	4.09	.61	.19	.04
		Hazard rate + poly	3	22148.43	0.00	.77	.18	.04
Truncate at 500 m	1980	Uniform + cosine	3	22234.13	185.62	.00	.30	.01
		Uniform + poly	3	22533.32	484.81	.00	.40	.01
		Half‐normal + cosine	3	22153.85	105.34	.00	.26	.02
		Half‐normal + Hermite	1	22503.44	454.93	.00	.36	.01
		Hazard rate + cosine	3	22050.69	2.18	.62	.19	.04
		Hazard rate + poly	3	22048.51	0.00	.70	.19	.04
Truncate at 480 m	1970	Uniform + cosine	3	22025.26	165.86	.00	.30	.01
		Uniform + poly	0	0.00	0.00	.00	.00	.00
		Half‐normal + cosine	3	21952.22	92.82	.00	.26	.02
		Half‐normal + Hermite	1	22278.42	419.02	.00	.36	.01
		Hazard rate + cosine	3	21863.44	4.04	.19	.20	.04
		Hazard rate + poly	3	21859.40	0.00	.37	.20	.04

Note

*n* = number of observations; Model (key) = Key function with series expansion; AIC = Akaike information criterion; ΔAIC = delta AIC value within comparable data selections; χ^2^ GOF (*p*) = chi‐square goodness of fit *p*‐value; P = detection probability function; P *cv* = detection probability coefficient of variation. We chose to use data truncated at 520 m with the hazard‐rate model and polynomial, for all analyses.

**TABLE A3:** Stratified distance sampling survey parameter estimates for habitat classes each year for Bleaklow and Margery Hill

	*n*	*L*	*n*/*L*	*n*/*L cv*	*n*/*L* LCL	*n*/*L* UCL	*K*	*E* (s)	*E* (s) *cv*	D̂	D̂ *cv*	D̂ LCL	D̂ UCL
AG17	11	8.3	1.3	0.61	0.3	5.3	7	2.31	0.25	16.0	0.67	3.9	64.8
GMB17	41	27.0	1.5	0.31	0.8	2.9	17	1.10	0.05	8.7	0.31	4.5	16.8
GMH17	12	9.8	1.2	0.31	0.5	2.9	5	1.28	0.12	8.2	0.34	3.5	18.8
RB17	93	19.8	4.7	0.32	2.3	9.4	11	1.19	0.03	29.3	0.32	14.4	58.8
UB17	145	47.1	3.1	0.17	2.2	4.4	24	1.16	0.02	18.7	0.17	13.0	26.7
UH17	2	8.7	0.2	1.12	0.0	1.8	9	1.00	0.00	1.2	1.12	0.1	9.6
AG18	20	8.7	2.3	0.55	0.7	8.1	7	1.16	0.10	14.0	0.56	4.0	49.1
GMB18	102	27.2	3.8	0.12	2.9	4.8	18	1.09	0.03	21.4	0.13	16.4	28.0
GMH18	32	9.6	3.3	0.23	1.6	7.0	4	1.25	0.06	21.8	0.24	10.7	44.1
RB18	99	19.9	5.0	0.20	3.2	7.7	12	1.11	0.02	28.9	0.20	18.5	45.0
UB18	239	47.1	5.1	0.10	4.1	6.3	23	1.15	0.02	30.5	0.11	24.3	38.6
UH18	12	8.9	1.3	0.62	0.4	5.0	10	1.56	0.11	11.0	0.63	3.0	40.9
AG19	19	8.0	2.4	0.50	0.7	7.6	7	1.17	0.09	14.5	0.51	4.5	46.8
GMB19	80	25.1	3.2	0.23	1.9	5.2	17	1.10	0.03	18.3	0.24	11.2	30.3
GMH19	12	9.1	1.3	1.04	0.1	14.2	5	1.23	0.10	8.5	1.04	0.8	89.9
RB19	86	18.3	4.7	0.19	3.0	7.3	10	1.16	0.03	28.5	0.20	18.2	44.7
UB19	196	43.6	4.5	0.14	3.3	6.1	23	1.12	0.02	26.3	0.15	19.1	36.3
UH19	8	8.1	1.0	0.73	0.2	4.4	9	1.04	0.11	5.4	0.74	1.2	24.2
AG20	14	8.9	1.6	0.39	0.6	4.0	7	1.09	0.06	9.0	0.40	3.5	22.8
GMB20	25	27.6	0.9	0.25	0.5	1.5	17	1.15	0.05	5.5	0.26	3.2	9.4
GMH20	10	10.0	1.0	0.42	0.3	3.7	4	1.00	0.00	5.2	0.42	1.4	19.0
RB20	150	20.0	7.5	0.30	3.8	14.7	11	1.07	0.01	42.0	0.31	21.4	82.5
UB20	193	47.5	4.1	0.24	2.4	6.7	23	1.04	0.01	22.1	0.25	13.2	36.9
UH20	10	8.9	1.1	0.48	0.4	3.2	9	1.00	0.00	5.9	0.48	2.0	17.0
AG21	11	8.4	1.3	0.56	0.4	4.5	8	1.49	0.12	10.2	0.57	3.0	35.7
GMB21	37	26.9	1.4	0.20	0.9	2.1	16	1.08	0.05	7.8	0.22	4.9	12.3
GMH21	13	9.8	1.3	0.46	0.4	4.6	5	1.15	0.14	8.0	0.49	2.4	26.7
RB21	116	19.7	5.9	0.24	3.4	10.2	10	1.11	0.03	34.2	0.25	19.8	59.6
UB21	192	47.1	4.1	0.14	3.0	5.5	24	1.15	0.02	24.5	0.15	17.9	33.9
UH21	5	10.5	0.5	0.91	0.1	2.8	10	0.82	0.18	2.0	0.93	0.3	12.0

Note

*n* = encounters; *L* = line length km; *K* = number of transects; *E*(s) = mean cluster size; D̂ = density estimate km^−2^; *cv* = parameter coefficient of variation; LCL & UCL = 95% confidence intervals. D̂ is calculated with probability density function *f*(0) = 0.010467 and *f*(0) *cv* = 0.0407 (Buckland et al., 2001, 84,85). AG = acid grassland; GMB = grouse moor bog; GMH = grouse moor heather; RB = restored bog; UB = unrestored bog; UH = unmanaged dwarf shrub heath and each year shown as suffix, for example, AG17 is acid grassland in 2017 survey.

**TABLE A4:** Pairwise *t*‐tests comparing strata based on values from Table 1

S1	S2	D̂ Difference	SE diff	*t* Stat	df *t* stat	*p* Value	Significant	Bonferonni‐corrected significant	Effect size
Habitats									
Acid grassland	Grouse moor bog	−0.26	3.2	0.08	66.04	.934			0.01
Acid grassland	Grouse moor heather	1.84	3.6	0.50	64.44	.615			0.06
**Acid grassland**	**Restored bog**	**−20.73**	**4.9**	**4.26**	**105.67**	.**000**	*	**	**0.38**
**Acid grassland**	**Unrestored bog**	**−12.44**	**3.3**	**3.75**	**86.17**	.**000**	*	**	**0.37**
**Acid grassland**	**Unmanaged heath**	**7.09**	**3.2**	**2.23**	**61.53**	.**029**	*		**0.27**
Grouse moor bog	Grouse moor heather	2.10	2.8	0.76	47.19	.449			0.11
**Grouse moor bog**	**Restored bog**	**−20.46**	**4.2**	**4.83**	**85.35**	.**000**	*	**	**0.46**
**Grouse moor bog**	**Unrestored bog**	**−12.18**	**2.3**	**5.28**	**313.71**	.**000**	*	**	**0.29**
**Grouse moor bog**	**Unmanaged heath**	**7.35**	**2.1**	**3.53**	**139.46**	.**001**	*	**	**0.29**
**Grouse moor heather**	**Restored bog**	**−22.57**	**4.6**	**4.89**	**90.60**	.**000**	*	**	**0.46**
**Grouse moor heather**	**Unrestored bog**	**−14.28**	**2.9**	**4.86**	**66.43**	.**000**	*	**	**0.51**
Grouse moor heather	Unmanaged heath	5.25	2.8	1.90	43.10	.064			0.28
Restored bog	Unrestored bog	8.29	4.3	1.92	99.03	.057			0.19
**Restored bog**	**Unmanaged heath**	**27.82**	**4.2**	**6.55**	**82.52**	.**000**	*	**	**0.58**
**Unrestored bog**	**Unmanaged heath**	**19.53**	**2.3**	**8.40**	**222.27**	.**000**	*	**	**0.49**
Comparison of habitats year to year									
AG17	AG18	2.1	13.1	0.15	14.11	.877			0.04
AG18	AG19	0.6	10.7	0.05	12.96	.957			0.01
AG19	AG20	5.6	8.2	0.67	9.30	.510			0.21
AG20	AG21	−1.2	6.8	0.18	12.43	.859			0.05
**GMB17**	**GMB18**	**−12.7**	**3.8**	**3.29**	**40.07**	.**002**	*	**	**0.46**
GMB18	GMB19	3.0	5.0	0.60	31.19	.549			0.11
**GMB19**	**GMB20**	**12.9**	**4.6**	**2.88**	**21.65**	.**009**	*	**	**0.53**
GMB20	GMB21	−2.3	2.1	1.09	35.64	.283			0.18
GMH17	GMH18	−13.6	5.9	2.31	5.59	.063			0.70
GMH18	GMH19	13.3	10.3	1.29	6.54	.239			0.45
GMH19	GMH20	3.3	9.1	0.35	4.58	.738			0.16
GMH20	GMH21	−2.8	4.4	0.62	7.28	.554			0.22
RB17	RB18	0.4	11.1	0.03	17.95	.975			0.01
RB18	RB19	0.4	8.0	0.05	22.45	.964			0.01
RB19	RB20	−13.5	13.8	0.97	14.28	.344			0.25
RB20	RB21	7.8	15.1	0.51	17.82	.612			0.12
**UB17**	**UB18**	**−11.9**	**4.5**	**2.64**	**57.93**	.**011**	*	**	**0.33**
UB18	UB19	4.2	4.9	0.86	55.74	.393			0.11
UB19	UB20	4.2	65.0	0.65	43.63	.518			0.10
UB20	UB21	−2.4	6.3	0.38	41.99	.705			0.06
UH17	UH18	−9.8	7.1	1.38	10.37	.195			0.39
UH18	UH19	5.6	8.1	0.69	15.80	.498			0.17
UH19	UH20	−0.5	4.9	0.10	15.09	.919			0.03
UH20	UH21	3.8	3.4	1.12	14.58	.279			0.28
Years (Bleaklow and Margery Hill combined)									
**2017**	**2018**	**−9.21**	**4.0**	**2.29**	**57.56**	.**025**	*		**0.29**
2018	2019	3.65	3.9	0.94	61.36	.350			0.12
2019	2020	3.13	5.4	0.58	47.34	.563			0.08
2020	2021	−0.36	5.6	0.06	49.37	.948			0.01
Survey areas 2019									
**Bleaklow**	**Margery Hill**	**8.68**	**6.8**	**1.27**	**23.31**	.**214**			**0.25**
**Bleaklow**	**Holme Moss**	**21.13**	**4.2**	**5.03**	**18.78**	.**000**	*	**	**0.76**
**Bleaklow**	**Peripheral Squares**	**21.10**	**4.1**	**5.20**	**16.90**	.**000**	*	**	**0.78**
Margery Hill	Holme Moss	12.45	5.8	2.13	14.76	.050			0.48
**Margery Hill**	**Peripheral Squares**	**12.42**	**5.7**	**2.16**	**13.89**	.**049**	*		**0.50**
Holme Moss	Peripheral Squares	−0.03	2.0	0.01	27.84	.987			0.00

Note

S1 = Stratum 1; S2 = Stratum 2. D̂ difference subtracts S2 D̂ from S1 D̂. A positive value indicates Stratum 1 is larger; a negative value means Stratum 2 is larger. SE is the standard error of D̂ difference. Values are assessed with Satterthwaite *t*‐test reporting *t*‐statistic and degrees of freedom. Asterisk * and bold lines indicate *p*‐value significant and using Bonferroni within‐cohort correction. Effect size calculated with Cohen’s *d* and considered as *r* = .10 (small); *r* = .30 (medium); *r* = .50 (large) (Field et al., 2012).

Abbreviations: AG, acid grassland; GMB, grouse moor bog; GMH, grouse moor heather; RB, restored bog; UB, unrestored bog; UDSH, unmanaged dwarf shrub heath.

**TABLE A5:** Pairwise *t*‐tests comparing habitat class strata each year based on values from Table A1 for Bleaklow and Margery Hill

Comparison between habitats within each year									
S1	S2	D̂ Difference	SE Diff	*t* Stat	df *t* stat	*p*‐Value	Significant	Bonferroni‐corrected significant	Effect size
AG17	GMB17	7.3	10.9	0.66	9.23	.521			0.21
AG17	GMH17	7.8	10.9	0.72	9.17	.492			0.23
AG17	RB17	−13.2	14.1	0.93	17.59	.360			0.22
AG17	UB17	−2.7	11.0	0.24	9.72	.810			0.08
AG17	UH17	14.8	10.7	1.39	8.37	.200			0.43
GMB17	GMH17	0.5	3.9	0.14	16.60	.891			0.03
GMB17	RB17	−20.5	9.8	2.09	12.31	.058			0.51
**GMB17**	**UB17**	**−9.9**	**4.2**	**2.34**	**43.72**	.**023**	*		**0.33**
**GMB17**	**UH17**	**7.5**	**3.1**	**2.44**	**23.74**	.**022**	*		**0.45**
GMH17	RB17	−21.0	9.8	2.14	12.17	.053			0.52
**GMH17**	**UB17**	**−10.5**	**4.2**	**2.48**	**22.51**	.**021**	*		**0.46**
GMH17	UH17	7.0	3.1	2.28	8.00	.052			0.63
RB17	UB17	10.6	9.9	1.06	13.11	.307			0.28
**RB17**	**UH17**	**28.0**	**9.5**	**2.94**	**10.92**	.**013**	*		**0.66**
**UB17**	**UH17**	**17.5**	**3.5**	**4.98**	**32.92**	.**000**	*	**	**0.66**
AG18	GMB18	−7.4	8.2	0.90	8.18	.393			0.30
AG18	GMH18	−7.9	9.4	0.83	9.99	.422			0.25
AG18	RB18	−14.9	9.7	1.53	13.63	.148			0.38
AG18	UB18	−16.6	8.4	1.97	9.00	.080			0.55
AG18	UH18	2.9	10.4	0.28	14.60	.782			0.07
GMB18	GMH18	−0.4	5.8	0.07	5.87	.940			0.03
GMB18	RB18	−7.5	7.5	1.17	17.67	.256			0.27
**GMB18**	**UB18**	**−9.1**	**4.1**	**2.22**	**55.33**	.**030**	*		**0.29**
GMB18	UH18	10.4	7.4	1.39	12.86	.186			0.36
GMH18	RB18	−7.1	7.8	12.61	12.61	.380			0.25
GMH18	UB18	−8.7	6.1	1.44	7.03	.193			0.48
GMH18	UH18	10.8	8.7	1.24	12.73	.235			0.33
RB18	UB18	−1.6	6.6	0.24	20.39	.806			0.05
RB18	UH18	17.9	9.1	1.97	20.27	.062			0.40
**UB18**	**UH18**	**19.5**	**7.6**	**2.56**	**14.43**	.**022**	*		**0.56**
AG19	GMB19	−3.8	8.5	0.44	11.14	.664			0.13
AG19	GMH19	6.1	11.5	0.52	9.00	.613			0.17
AG19	RB19	−14.0	9.2	1.51	13.28	.153			0.38
AG19	UB19	−11.8	8.3	1.42	10.31	.184			0.40
AG19	UH19	9.2	8.4	1.09	10.09	.300			0.32
GMB19	GMH19	9.9	9.8	1.00	6.17	.354			0.37
GMB19	RB19	−10.2	7.0	1.46	21.70	.158			0.30
GMB19	UB19	−8.0	5.7	1.41	40.21	.166			0.22
**GMB19**	**UH19**	**13.0**	**5.8**	**2.22**	**24.00**	.**036**	*		**0.41**
GMH19	RB19	−20.0	10.4	1.91	7.52	.094			0.57
GMH19	UB19	−17.9	9.6	1.85	5.76	.115			0.61
GMH19	UH19	3.1	9.7	0.32	5.77	.760			0.13
RB19	UB19	2.2	6.6	0.32	20.71	.746			0.07
**RB19**	**UH19**	**23.2**	**68.0**	**3.40**	**17.78**	.**003**	*	**	**0.63**
**UB19**	**UH19**	**21.0**	**5.5**	**3.82**	**24.96**	.**001**	*	**	**0.61**
AG20	GMB20	3.5	3.8	0.92	8.51	.381			0.30
AG20	GMH20	3.7	4.2	0.89	9.39	.393			0.28
**AG20**	**RB20**	**−33.0**	**13.1**	**2.51**	**11.96**	.**027**	*		**0.59**
**AG20**	**UB20**	**−13.1**	**6.4**	**2.05**	**28.48**	.**049**	*		**0.36**
AG20	UH20	3.1	4.5	0.68	13.00	.507			0.19
GMB20	GMH20	0.2	2.6	0.08	5.88	.930			0.03
**GMB20**	**RB20**	**−36.5**	**12.7**	**2.87**	**10.64**	.**016**	*		**0.66**
**GMB20**	**UB20**	**−16.7**	**5.5**	**3.02**	**26.49**	.**006**	*	**	**0.51**
GMB20	UH20	−0.4	3.1	0.13	12.28	.894			0.04
**GMH20**	**RB20**	**−36.8**	**12.8**	**2.86**	**10.99**	.**015**	*		**0.65**
**GMH20**	**UB20**	**−16.9**	**5.8**	**2.92**	**26.21**	.**007**	*	**	**0.50**
GMH20	UH20	−0.6	3.6	0.18	10.59	.860			0.06
RB20	UB20	19.9	13.7	1.45	14.25	.168			0.36
**RB20**	**UH20**	**36.1**	**12.9**	**2.79**	**11.41**	.**017**	*		**0.64**
**UB20**	**UH20**	**16.2**	**6.0**	**2.68**	**31.19**	.**011**	*		**0.43**
AG21	GMB21	2.4	6.1	0.40	8.94	.690			0.13
AG21	GMH21	2.2	7.0	0.31	12.19	.756			0.09
**AG21**	**RB21**	**−24.0**	**10.2**	**2.36**	**16.68**	.**031**	*		**0.50**
AG21	UB21	−14.3	6.8	2.10	14.13	.054			0.49
AG21	UH21	8.2	6.2	1.32	9.34	.216			0.40
GMB21	GMH21	−0.2	4.2	0.05	6.66	.961			0.02
**GMB21**	**RB21**	**−26.4**	**8.5**	**3.12**	**10.56**	.**010**	*		**0.69**
**GMB21**	**UB21**	**−16.8**	**3.9**	**4.34**	**38.25**	.**000**	*	**	**0.57**
**GMB21**	**UH21**	**5.7**	**2.5**	**2.29**	**22.79**	.**032**	*		**0.43**
**GMH21**	**RB21**	**−26.2**	**9.1**	**2.86**	**13.19**	.**013**	*		**0.62**
**GMH21**	**UB21**	**−16.6**	**5.2**	**3.18**	**15.04**	.**006**	*	**	**0.63**
GMH21	UH21	5.9	4.3	1.38	7.42	.208			0.45
RB21	UB21	9.7	9.0	1.08	13.62	.300			0.28
**RB21**	**UH21**	**32.2**	**8.5**	**3.77**	**10.81**	.**003**	*	**	**0.75**
**UB21**	**UH21**	**22.5**	**4.0**	**5.61**	**37.44**	.**000**	*	**	**0.68**

Note

S1 = Stratum 1; S2 = Stratum 2. D̂ difference subtracts S2 D̂ from S1 D̂. A positive value indicates Stratum 1 is larger; a negative value means Stratum 2 is larger. SE is the standard error of D̂ difference. Values are assessed with Satterthwaite *t*‐test reporting *t*‐statistic and degrees of freedom. Asterisk * and bold lines indicate *p*‐value significant and also when applying Bonferonni within‐cohort correction. AG = acid grassland; GMB = grouse moor bog; GMH = grouse moor heather; RB = restored bog; UB = unrestored bog; UH = unmanaged dwarf shrub heath and each year shown as suffix, for example, AG17 is acid grassland in 2017 survey.

**TABLE A6:** Abundance of mountain hares for Peak District for year 2019, based on density estimates derived from pooled observations for each of the four denoted surveyed areas

	Bleaklow	Margery Hill	Holme Moss	Peripheral areas	
Density km^−2^	27.4	18.6	6.1	6.2	
Density LCL	19.9	9.7	3.4	4.1	
Density UCL	37.8	35.5	10.9	9.4	
					Total
Area km^2^	40.4	40.4	40.4	236.3	357.5
Abundance	1107	750	247	1458	3562
Abundance LCL	802	393	139	957	2291
Abundance UCL	1528	1433	442	2221	5624

Note

Calculation of km^2^ for each surveyed areas is based on relevant habitat classes only, that is, acid grassland; grouse moor bog; grouse moor heather; restored bog; unrestored bog; and unmanaged dwarf shrub heath. Thus non‐relevant types, for example, woodland are excluded. Density estimate is shown with 95% confidence limits; abundance also with 95% confidence limits.
